# Targeting genome integrity dysfunctions impedes metastatic potency in non–small cell lung cancer circulating tumor cell–derived explants

**DOI:** 10.1172/jci.insight.155804

**Published:** 2022-06-08

**Authors:** Tala Tayoun, Vincent Faugeroux, Marianne Oulhen, Olivier Déas, Judith Michels, Laura Brulle-Soumare, Stefano Cairo, Jean-Yves Scoazec, Virginie Marty, Agathe Aberlenc, David Planchard, Jordi Remon, Santiago Ponce, Benjamin Besse, Patricia L. Kannouche, Jean-Gabriel Judde, Patrycja Pawlikowska, Françoise Farace

**Affiliations:** 1 Gustave Roussy, Paris-Saclay University, “Circulating Tumor Cells” Translational Platform, CNRS UMS3655 – INSERM US23 AMMICA, Villejuif, France.; 2 INSERM, U981 “Identification of Molecular Predictors and new Targets for Cancer Treatment”, Villejuif, France.; 3 XenTech, Evry, France.; 4 Gustave Roussy, Paris-Saclay University, Department of Cancer Medicine, Villejuif, France.; 5 Gustave Roussy, Paris-Saclay University, “Histo-Cytopathology” Translational Platform, CNRS UMS3655 – INSERM US23 AMMICA, Villejuif, France.; 6 Department of Medical Oncology, Clara Campal Comprehensive Oncology Center (HM-CIOCC), Hospital HM New Delphi, HM Hospitals, Barcelona, Spain.; 7 Paris-Saclay University, CNRS UMR9019 “Genome Integrity and Cancers”, Gustave Roussy, Villejuif, France.

**Keywords:** Oncology, Lung cancer, Mouse models

## Abstract

DNA damage and genomic instability contribute to non–small cell lung cancer (NSCLC) etiology and progression. However, their therapeutic exploitation is disappointing. CTC-derived explants (CDX) offer systems for mechanistic investigation of CTC metastatic potency and may provide rationale for biology-driven therapeutics. Four CDX models and 3 CDX-derived cell lines were established from NSCLC CTCs and recapitulated patient tumor histology and response to platinum-based chemotherapy. CDX (GR-CDXL1, GR-CDXL2, GR-CDXL3, GR-CDXL4) demonstrated considerable mutational landscape similarity with patient tumor biopsy and/or single CTCs. Truncal alterations in key DNA damage response (DDR) and genome integrity–related genes were prevalent across models and assessed as therapeutic targets in vitro, in ovo, and in vivo. GR-CDXL1 presented homologous recombination deficiency linked to biallelic *BRCA2* mutation and *FANCA* deletion, unrepaired DNA lesions after mitosis, and olaparib sensitivity, despite resistance to chemotherapy. SLFN11 overexpression in GR-CDXL4 led to olaparib sensitivity and was in coherence with neuroendocrine marker expression in patient tumor biopsy, suggesting a predictive value of SLFN11 in NSCLC histological transformation into small cell lung cancer (SCLC). Centrosome clustering promoted targetable chromosomal instability in GR-CDXL3 cells. These CDX unravel DDR and genome integrity–related defects as a central mechanism underpinning metastatic potency of CTCs and provide rationale for their therapeutic targeting in metastatic NSCLC.

## Introduction

Advanced non–small cell lung cancer (NSCLC) has a poor prognosis, owing to severe metastatic spread and acquired treatment resistance, with no curative therapy (1). Over the past 2 decades, genomic profiling has become a standard diagnostic tool in NSCLC and the implementation of targeting oncogenic alterations of tyrosine kinases (e.g., epidermal growth factor receptor [EGFR], anaplastic lymphoma kinase [*ALK*] or c-ros oncogene 1 [*ROS1*] fusions) has demonstrated unprecedented clinical benefits in corresponding patient subsets (2). In the majority of advanced-stage NSCLC lacking targetable mutations, platinum-based chemotherapy remains a cornerstone in first-line treatment, with or without immunotherapy (3). DNA damage and genome instability in response to mutagenic insults in NSCLC have received considerable attention and represent an attractive therapeutic target (4). Synthetic lethality approaches to cancer therapy and the clinical development of poly(ADP-ribose) polymerase inhibitors (PARPi) have been a major breakthrough in the treatment of *BRCA1/2*-mutant cancers (5, 6) (ovary, breast, pancreas, and prostate) and are highly effective in potentiating chemotherapy, based on the biological rationale that the deficiency in DNA repair machinery modulates tumor response to platinum chemotherapy (7–10). However, clinical studies assessing PARPi efficacy either in combination with chemotherapy or as maintenance treatment have failed to yield any significant benefit in chemosensitive NSCLC tumors with or without *BRCA* mutations (11, 12).

Genome integrity is constantly threatened by different types of DNA lesions, with DNA double-stranded breaks (DSBs) being the most cytotoxic and mainly repaired by homologous recombination (HR) (13). Failure to resolve DSBs owing to loss-of-function mutations in genes encoding key players in the DNA damage response (DDR) — such as *BRCA1*, *BRCA2* — and HR deficiency (HRD) has paved the way toward DDR-directed therapeutic strategies in several cancers (6). Although *BRCA* mutations are not very common in NSCLC, *BRCAness* — a state of defect in the DNA repair machinery mimicking *BRCA1*/*BRCA2* loss — has been reported several times in NSCLC. However, its therapeutic implications remain to be elucidated (14). To this end, in-depth mechanistic understanding of DDR mechanisms contributing to genome stability maintenance is crucial to evaluate their impact in NSCLC and offer greater DDR-based therapeutic windows.

The main route to metastatic spread is by circulating tumor cell (CTC) dissemination from the primary tumor and/or distinct metastatic foci (15). CTC count is a negative prognostic marker in several malignancies, including NSCLC (16–19). The metastasis-initiating capacity of a minor subset of patient CTCs with cancer stem cell properties has been reported in immunodeficient mice and CTC-derived explant (CDX) models were established in different cancer types (20–24). CDX may provide relevant insight into the biology of metastasis, serve to examine mechanisms underpinning metastatic disease, and provide a platform to decipher biology-driven treatment strategies (25). Nevertheless, their development remains extremely difficult due to CTC paucity in the bloodstream and technical challenges related to their phenotypic heterogeneity (26). In NSCLC, only one CDX model has been generated to date (22).

Here, we hypothesized that genome integrity-related dysfunctions are critical processes in CTC metastatic potency and NSCLC progression. CDX models can offer a platform for the functional characterization of DDR and genome integrity maintenance mechanisms, as well as the testing of biology-driven therapeutic hypotheses. We report the development of 4 NSCLC CDX models and 3 in vitro CDX-derived cell lines, which recapitulate patient tumor pathology and chemoresponse. Genomic analysis of the CDX, cell line, their matching tumor biopsy (TB), and patient single CTCs indicated an important overlap of mutational landscapes. Phylogenetic reconstruction revealed clonal alterations in genes involved in the DDR and genome integrity across all models. Through mechanistic analysis, we detected HRD in GR-CDXL1 — concordant with biallelic somatic *BRCA2* mutation and *FANCA* deletion — and GR-CDXL4 cell lines. Both responded to PARPi olaparib treatment, consistent with SLFN11 overexpression in GR-CDXL4 cells, which has been described as a predictor of sensitivity to PARPi in several cancers (27, 28). On the other hand, mitotic defects with centrosome clustering events were prevalent in GR-CDXL3 and successfully assessed as therapeutic targets in vitro, in ovo, and in vivo. Our results open up perspectives for a therapeutic exploitation of the DDR and genome stability maintenance defects, as well as for predictive biomarker identification in metastatic NSCLC.

## Results

### Patient donors and establishment of NSCLC CDX models.

**Figure 1:**
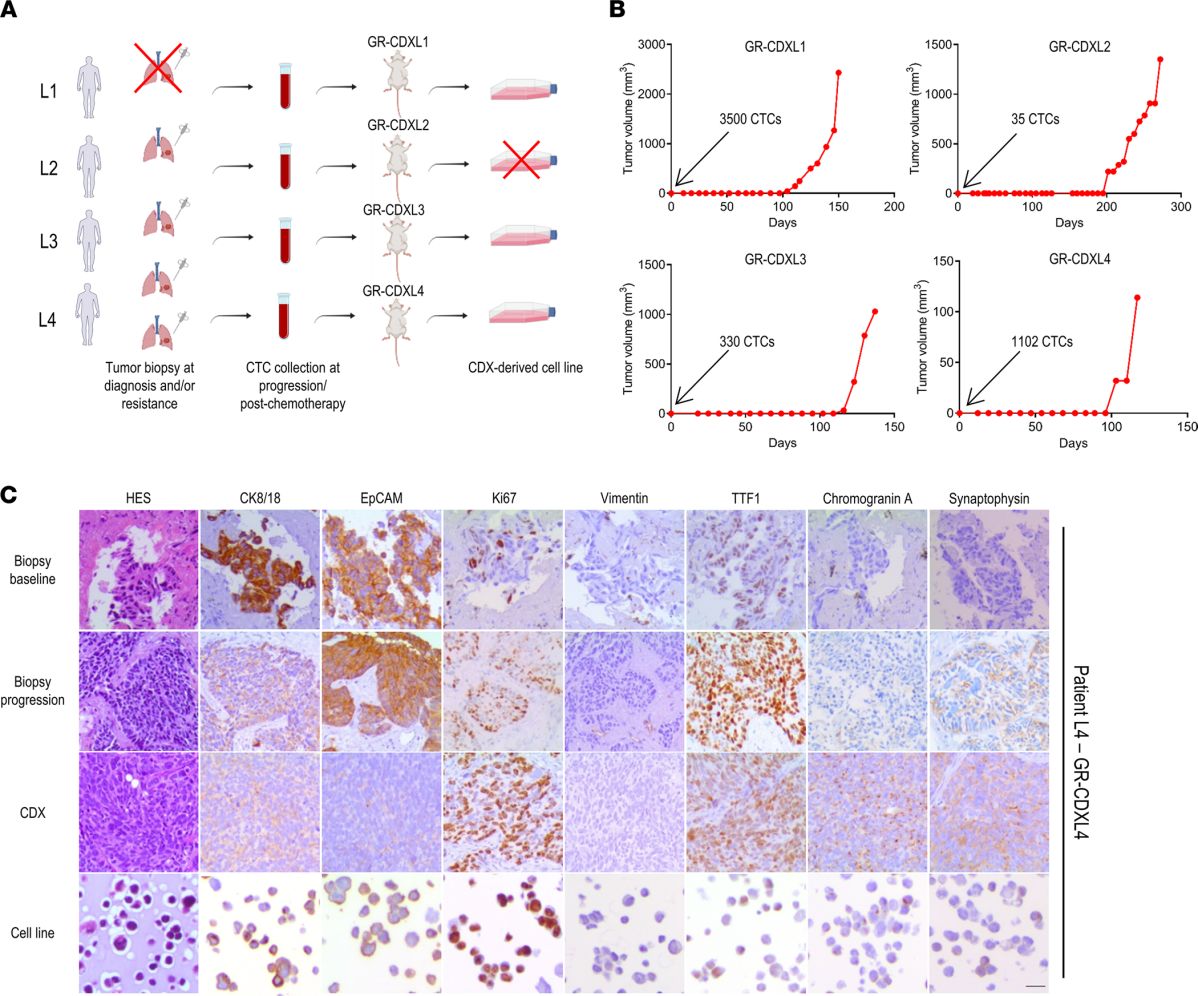
Establishment and characterization of CDX and CDX-derived cell lines. (**A**) Schematic of available patient samples and established CDX and CDX-derived cell lines (red cross = not available). (**B**) CDX tumor growth curves. Indicated number of CTCs was injected in NSG mice. Palpable CDX tumors were obtained after 100, 200, 116, and 100 days in GR-CDXL1, GR-CDXL2, GR-CDXL3, and GR-CDXL4, respectively. (**C**) IHC characterization of patient L4 TB at baseline and disease progression, GR-CDXL4 CDX tumor and CDX-derived cell line. Representative images of HES, CK8/18, EpCAM, Ki67, Vimentin, TTF1, Chromogranin A, and Synaptophysin stainings are shown at a total magnification of ×200. Scale bar: 10 μm.

Blood samples were collected from 55 patients with advanced NSCLC. Among them, 82% presented adenocarcinoma, and all but 10 patients were smokers (Table 1 and Supplemental Table 1; supplemental material available online with this article; https://doi.org/10.1172/jci.insight.155804DS1). After hematopoietic blood cell depletion, the CTC-enriched fraction was implanted into NSG (NOD.Cg-*Prkdc^scid^Il2rg^tmlWjl^*/SzJ) mice. The average number of EpCAM^+^ CTCs detected by the CellSearch system in a 7.5 mL blood sample was 163 with a median of 2 CTCs (range, 0–3903 CTCs). The average number of implanted epithelial CTCs (EpCAM^+^/cytokeratin^+^, CD45^–^) estimated based on CellSearch CTC counts was 693 with a median of 9 CTCs (range, 0–17694 CTCs). CTCs from patients P8 (termed L1), P37 (termed L2), P48 (termed L3), and P50 (termed L4) (3 adenocarcinomas, 1 squamous cell carcinoma) successfully generated 4 CDX tumors in mice called GR-CDXL1, GR-CDXL2, GR-CDXL3, and GR-CDXL4, respectively (Figure 1, A and B). Patients L1, L3, and L4 had high EpCAM^+^/pan-cytokeratin^+^ CTC counts (750, 177, and 243 CTCs per 7.5 mL blood, respectively) at the moment of implantation, while patient L2 presented only 10 CTCs (Supplemental Table 1). Indeed, 3500, 330, and 1102 CTCs were implanted in NSG mice for the establishment of GR-CDXL1, GR-CDXL3, and GR-CDXL4, respectively, while only 35 CTCs were injected in the case of GR-CDXL2 (Figure 1B). CDX were generated at a single time point, after first-line therapy (GR-CDXL3, GR-CDXL4) or second-line therapy (GR-CDXL1, GR-CDXL2). All 4 patients had unresected tumors, and their clinical history is summarized in Supplemental Figure 1. A TB was obtained at diagnosis for patients L2, L3, and L4. An additional TB at disease progression (at the time of CTC injection) was obtained for patient L4 (Figure 1A). Patient L2 had a *KRAS*-mutated tumor, and patient L4 had a *MET*-amplified tumor, while no known oncogenic driver alteration was detected for patient L3 (Supplemental Table 1 and Supplemental Figure 2A). Unfortunately, tumor cell content in patient L1 TB was not sufficient to perform routine molecular diagnosis.

At CDX passage 1, human origin of the tumor was validated by FISH testing of Alu element genetic marker (data not shown). Tumor fragments were used for CDX propagation in successive generations of NSG mice. Histopathology of the 4 CDX was assessed in comparison with available corresponding TB specimens. All TB and CDX tumors were of epithelial origin (positive for EpCAM and/or CK8/18), and none of the CDX expressed vimentin (Figure 1C and Supplemental Figure 2C). Poorly differentiated lung adenocarcinoma cells were detected in GR-CDXL1, GR-CDXL2 (Supplemental Figure 2C), and GR-CDXL4 (Figure 1C) tumors by HES stain, while in patient L3, TB 10% of tumor cells were positive for p40 and CK5/6 squamous markers (Supplemental Figure 2C). GR-CDXL1 CDX expressed neuroendocrine markers chromogranin A and synaptophysin in 10% of the cells (Supplemental Figure 2C). Foci of neuroendocrine cells expressing chromogranin A were detected in patient L4 diagnostic TB (10%), in TB at progression (20%), and in 50% and 30% of tumor cells in the CDX and the cell line, respectively. Synaptophysin was not expressed in patient L4 diagnostic TB, while it was detected in 60% of tumor cells in the TB at progression, in 40% of CDX tumor cells, and in 25% of the cell line (Figure 1C).

To evaluate whether CDX mimic patient responses to chemotherapy, in vivo drug assays were conducted in NSG mice (Supplemental Figure 3). GR-CDXL1 was resistant to cisplatin, mirroring patient L1 clinical progression at 2 months, while GR-CDXL2 showed tumor regression, recapitulating corresponding patient response to platinum salts (Supplemental Figure 1). In GR-CDXL3, the CDX tumor slowly progressed over the course of cisplatin treatment, similarly to patient L3, who progressed after 6 cycles of chemotherapy combination. In GR-CDXL4, cisplatin treatment resulted in delayed CDX tumor growth before progression after day 20, in accordance with patient L4’s partial response after 4 cycles of chemotherapy (Supplemental Figure 1). Paclitaxel treatment promoted tumor stabilization in GR-CDXL1, in accordance with a stable disease in patient L1. GR-CDXL2 exhibited tumor stabilization before sudden progression, which reflects patient initial tolerance to paclitaxel over a 2-month treatment course followed by disease progression. The GR-CDXL4 tumor was a nonresponder (Supplemental Figures 1 and 3). Overall, chemoresponse of the 4 CDX tumors mirrored that of corresponding patient tumors, which validated our models.

### Establishment of CDX-derived cell lines and in vivo and in ovo metastatic modeling.

**Figure 2:**
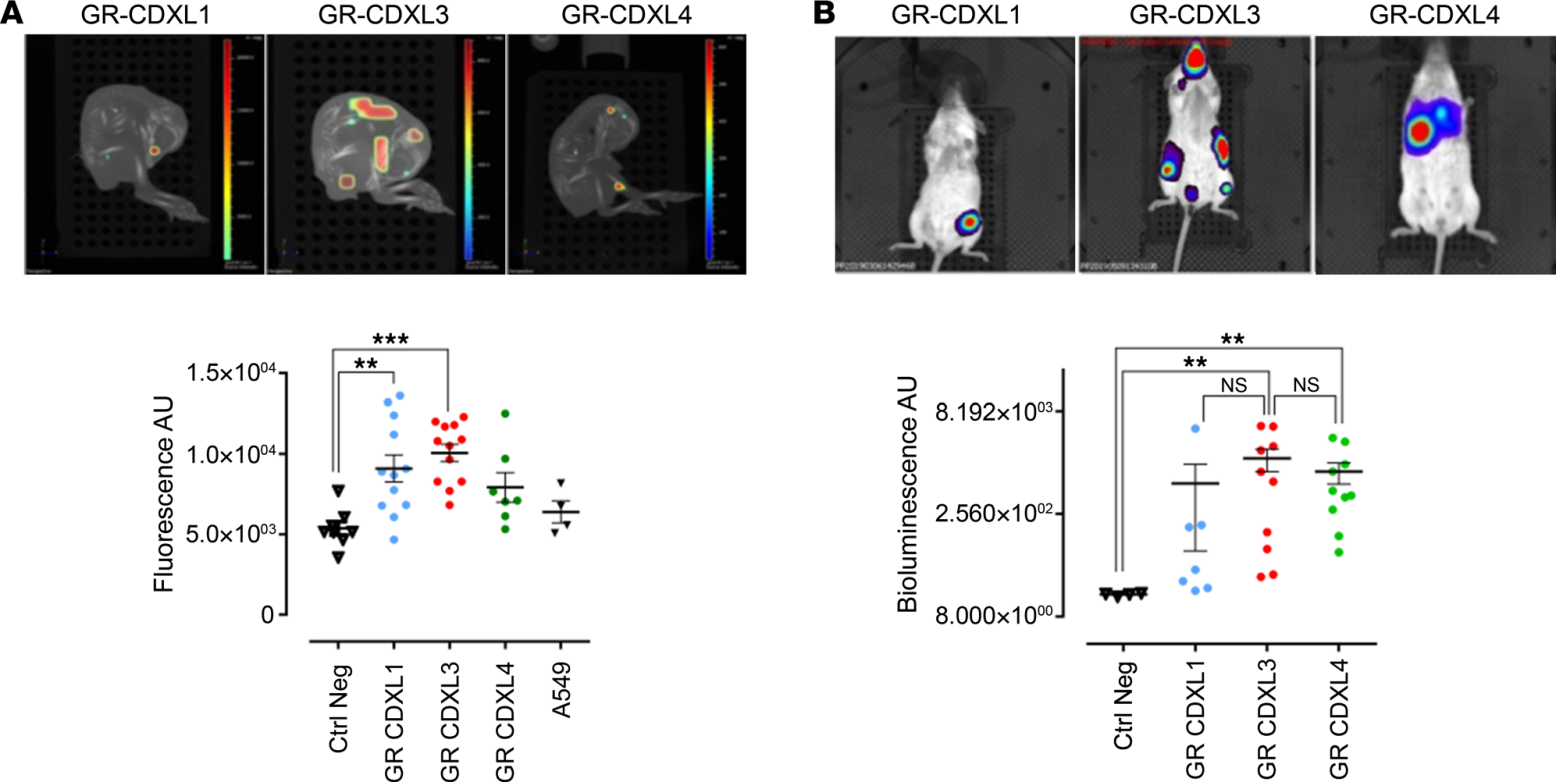
Evaluation of CDX-derived cell line metastatic potency in ovo and in vivo. (**A**) Metastatic capacity of CDX-derived cell lines in the CAM. mCherry-expressing CDX-derived cells were implanted into the CAM, and metastatic fluorescent signal was analyzed at day 7. Representative fluorescence/CT images of GR-CDXL1–, GR-CDXL3–, and GR-CDXL4–generated tumors are shown (top). Quantitative analysis of average fluorescence intensity (bottom); each point represents a single embryo. (**B**) Metastatic capacity of CDX-derived cell lines in NSG mice. Luciferase-expressing CDX-derived cells were grafted into NSG mice by IC to generate metastases. Representative BLI images of GR-CDXL1–, GR-CDXL3–, and GR-CDXL4–generated tumors are shown (top). Quantitative analysis of average BLI intensity (bottom); each point represents a single mouse. Data are mean ± SEM; **P ˂ 0.01, ***P < 0.001 by Kruskall Wallis and post hoc Dunn’s test.

At passage 2, CDX tumors were dissociated and human tumor cells were cultured in vitro. GR-CDXL1 and GR-CDXL4 cells grew as adherent microspheres, while GR-CDXL3 formed an adherent monolayer (Supplemental Figure 2B). Despite several attempts of cell expansion at different passages, GR-CDXL2 cells did not grow in vitro. Overall, 3 permanent CDX-derived cell lines were established with an average doubling time of 4 days. They all expressed a phenotype similar to their corresponding CDX (Figure 1C and Supplemental Figure 2C). Interestingly, CDX-derived cells expressed stem cell markers CD133 and CD166. ABCG2 and ALDH activity were detected in GR-CDXL1 cells, while CD90 was expressed by GR-CDXL1 and GR-CDXL3 cells (Supplemental Figure 2D). To assess the tumorigenicity and metastatic capacity of CDX-derived cell lines, we reinjected CDX-derived cells in the chorioallantoic membrane (CAM) of the chick embryo and NSG mice. For in ovo experiments, cells were infected with mCherry-expressing retroviral particles before engraftment on the CAM, as previously reported (29). The 3 CDX-derived cell lines formed tumor nodules, and GR-CDXL3 cells showed increased disseminating capacity compared with GR-CDXL1 and GR-CDXL4 (Figure 2A). To evaluate later stages of metastatic spread, intracardiac (IC) injection of luciferase-expressing cells was performed in NSG mice. All CDX-derived cell lines were tumorigenic. GR-CDXL1 cells formed localized single tumors, while GR-CDXL3 and GR-CDXL4 cells seeded multiple metastases (Figure 2B and Supplemental Figure 4).

### Genome characterization and phylogenetic analysis of TB, CTCs, CDX, and CDX-derived cell lines.

**Figure 3:**
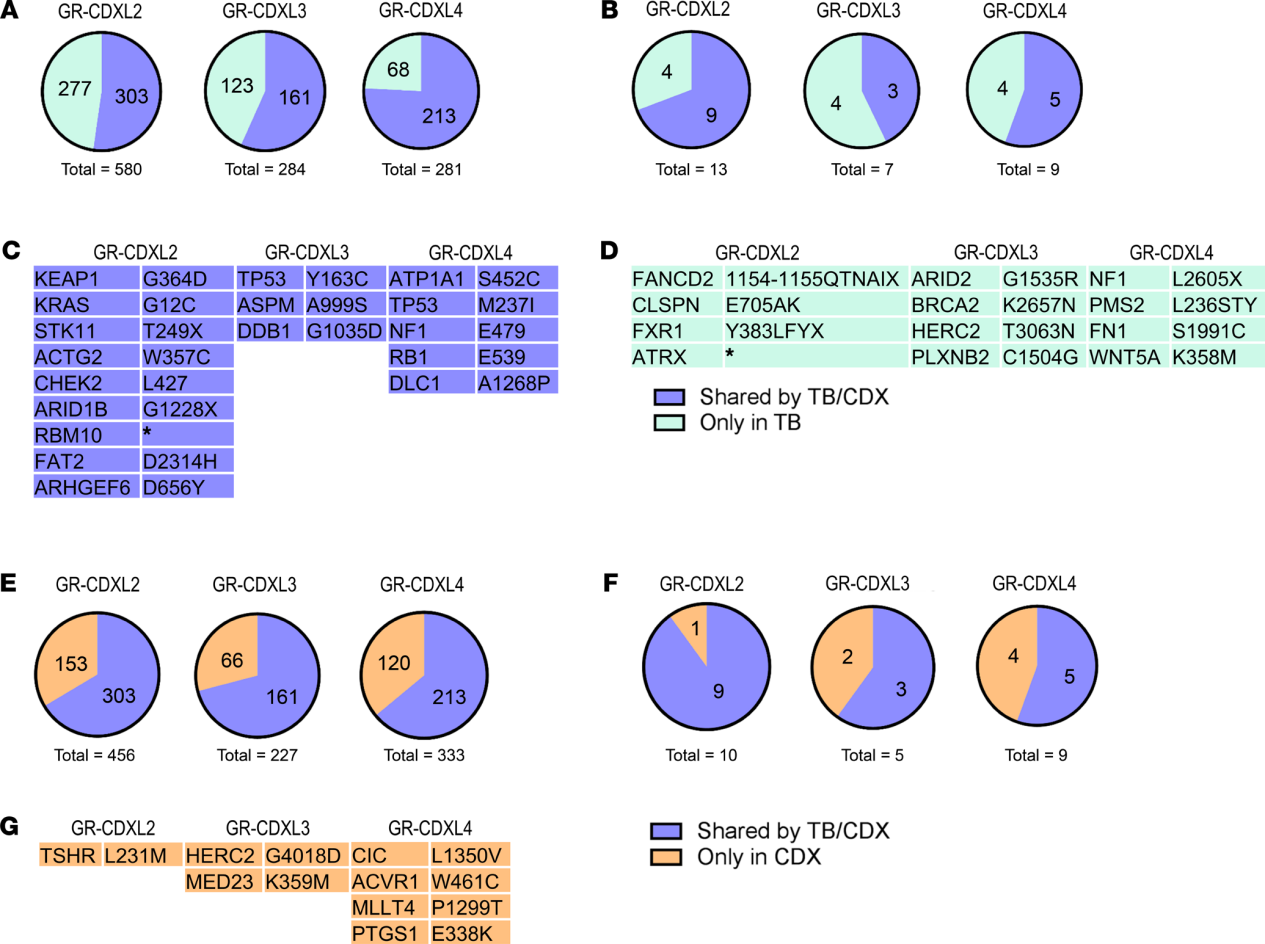
Comparative genomic analysis of biopsies and the CDX. (**A**) Fraction of TB mutations detected and undetected in the CDX. (**B**) Fraction of TB driver mutations detected and undetected in the CDX. (**C**) Mutated driver genes and their amino acid sequence variation in the biopsy and the CDX. (**D**) Mutated driver genes and amino acid sequence variation in the TB only. (**E**) Fraction of CDX mutations issued or not from the TB. (**F**) Fraction of CDX driver mutations issued or not from the TB. (**G**) Mutated driver genes and amino acid sequence variation in the CDX only.

To determine the extent to which the CDX is representative of the TB and evaluate genome alterations potentially associated with tumorigenic activity, we performed whole-exome sequencing (WES) analysis of TB of patients L2 and L3 at diagnosis and patient L4 TB at progression; we analyzed single CTCs from patients L1 and L3, and we analyzed the CDX models and the CDX-derived cell lines. Due to the lower quality of collected material, patient L4 diagnostic TB was excluded from WES analysis, and material was conserved for IHC. Single CTCs with satisfactory whole genome amplification quality controls could not be obtained for patients L2 and L4. All samples submitted for sequencing are annotated in Supplemental Figure 5. All WES data are available in Supplemental Data Sets 1–6. Sequencing depth, coverage, and number of variants identified in the TB, CTCs, CDX, and CDX-derived cell lines are provided in Supplemental Table 2 and Supplemental Table 3. In total, 52.24% (303 of 580) of mutations detected in the patient L2 TB specimen were found in the CDX (Figure 3A). Amino acid sequence variation of driver genes in the different samples is listed in Figure 3C. Patient L2 TB and CDX presented a *KRAS*-mutant tumor with concurring mutations in genes *KEAP1*, *STK11*, and *RBM10*. Driver mutation in the cell cycle checkpoint kinase and DDR gene *CHEK2* was also found in these samples (Figure 3, B and C). In total, 56.7% (161 of 284) of patient L3 TB alterations were conserved in GR-CDXL3 CDX, including driver *TP53* and DNA repair gene *DDB1* mutations (Figure 3, A, C, and D). A total of 75.8% (213 of 281) of mutations detected in patient L4 TB at progression was found in GR-CDXL4 CDX, including loss-of-function in tumor suppressor genes *RB1*, *TP53*, and *NF1* (Figure 3A). Overall, the important overlap of mutations between the CDX and the corresponding TB validates the clinical relevance of the CDX models. Importantly, these data reveal aberrations in genes involved in genome integrity maintenance through DNA repair mechanisms and the DDR.

Statistics of allele drop-out and false-positive rate of patients L1 and L3 single CTCs are shown in Supplemental Figure 11. Using variant calling criteria (present in at least 1 other tumor sample), a set of 24 unique variants with high variant allele frequencies was identified in L1-CTC1. All mutations were conserved in GR-CDXL1 CDX (Supplemental Table 3). Five single CTCs from patient L3 were analyzed by WES, and *TP53* driver mutation was recurrent in CTCs 1, 2, and 5; the TB; and the CDX. *ASPM*, a gene involved in mitotic spindle formation, was mutated in CTCs 2, 3, and 5; the TB; and the CDX, while a driver mutation in *DDB1* was detected in CTC5, the TB, and the CDX.

We then focused on the mutational profiles of the CDX, as these models may not only recapitulate primary tumor molecular characteristics, but also help track metastatic disease through tumorigenic CTCs. We therefore examined the mutations exclusive to the CDX (not in the corresponding TB), which may be potentially acquired during metastatic progression. In GR-CDXL2, 33.5% (153 of 456) of the detected mutations were exclusive to the CDX. In GR-CDXL3, 29% (66 of 227) of mutations were found in the CDX and the cell line (Figure 3E), including driver mutations in *ERBB2* and *MED23* (Figure 3, F and G). Finally, 36% (120 of 333) of mutations found in GR-CDXL4 CDX were exclusive to the CDX and the cell line (Figure 3, E–G). Overall, in all CDX models, approximately 30% of mutations were likely acquired during metastatic progression.

To evaluate the relevance of our CDX-derived cell lines, we performed a comparative genomic analysis with the corresponding CDX. The GR-CDXL1 CDX mutational profile presented 91.4% similarity with the cell line, including driver mutations in genes involved in DNA repair such as *ATRX*, *BRCA2*, and *TP53BP1*, along with chromatin remodeling genes, including *ARID1A* and *ARID1B* (Supplemental Figure 6, A–C). GR-CDXL3 and GR-CDXL4 CDX had 81.9% and 72% mutational overlap, respectively, with their corresponding cell line (Supplemental Figure 6A). These results reveal important mutational landscape similarities between the CDX and the CDX-derived cell line, as identified through hierarchical clustering of all variant genes (Supplemental Figure 7), thus validating our models of study.

**Figure 4:**
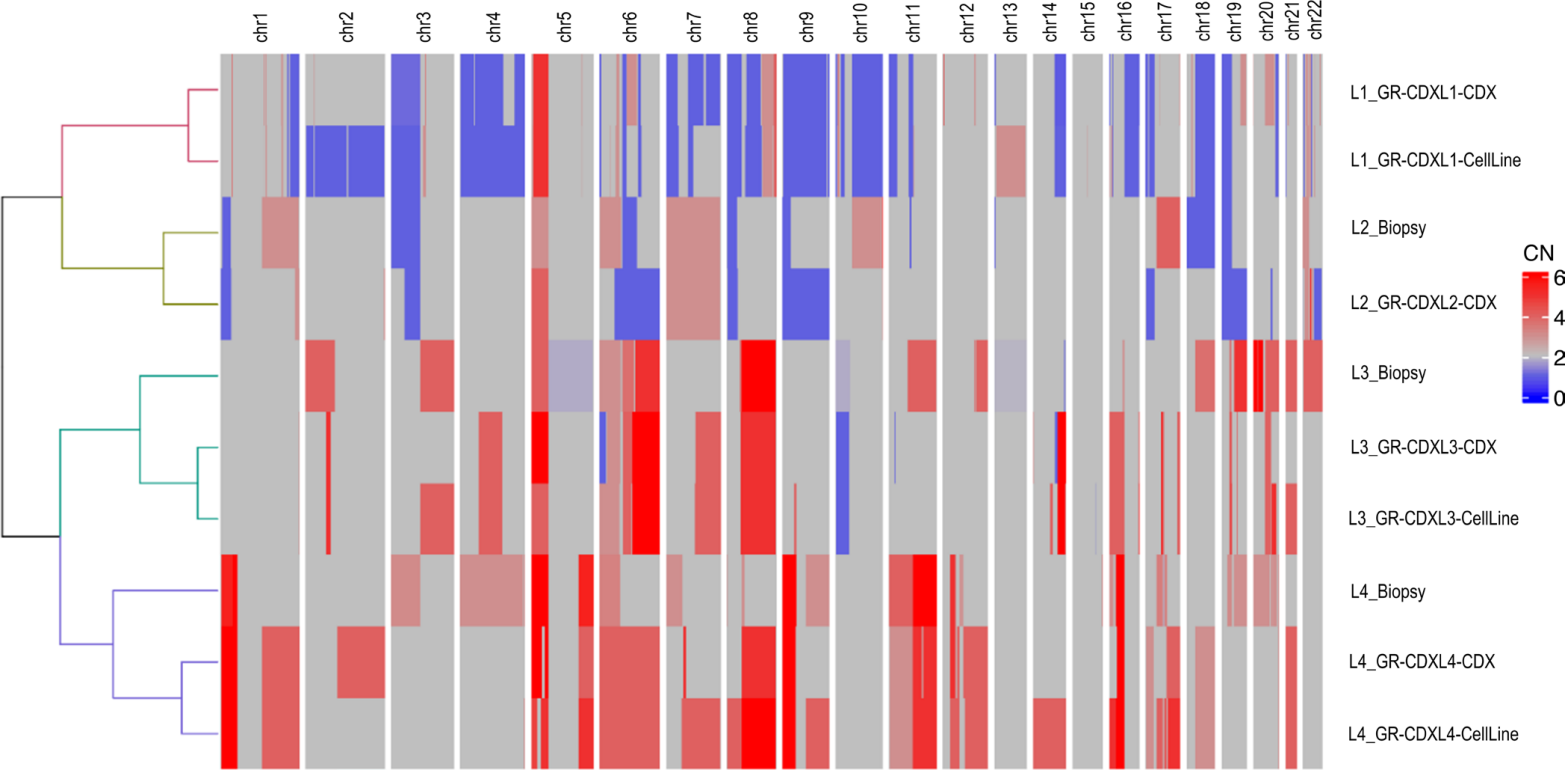
Heatmap of the CNA analysis of the TB, the CDX, and the CDX-derived cell lines. Unsupervised hierarchical clustering of CNA profiles was performed. Copy gains are shown in red, and copy losses are shown in blue. CN, copy number.

Next, we performed copy number alteration (CNA) analysis and examined shared alterations between TB specimen, the CDX, and the CDX-derived cell line for each model. Multiple CNAs were detected across the 4 models, highlighting chromosomal instability (CIN) (Figure 4 and Supplemental Figures 8 and 9). Unsupervised hierarchical clustering of all CNAs identified a single cluster in patients L1 samples, composed of the CDX and the cell line, and a single cluster in patient L2 samples, consisting of the patient TB and the CDX, with predominant copy number losses. On the other hand, 2 separate clusters were depicted among each sample from patients L3 and L4; the first was composed of the TB, and the second was composed of the CDX and the cell line, with predominant copy number gains (Figure 4). As commonly observed in lung cancer, loss of tumor suppressor genes *TP53* and *MAP2K4* and gain of *TERT* were identified in all samples; *PTEN* and *APC* losses were detected in GR-CDXL1 and GR-CDXL4 tumor samples and in GR-CDXL3 CDX and its cell line. Moreover, *RB1* loss was detected in GR-CDXL3 and GR-CDXL4, while *STK11* loss was detected in GR-CDXL1 and GR-CDXL2 samples. Several unstable chromosomal regions occurring across CDX models included DNA repair and DDR-related genes. Notably, the deletion of *FANCA* promoter (segment 16q24.3) in GR-CDXL1 samples (Supplemental Figure 8), *FHIT* loss (GR-CDXL1, GR-CDXL2, and GR-CDXL3 samples), *ARID1A* loss (GR-CDXL2 and GR-CDXL3 samples), and proto-oncogene *MDM4* gain (GR-CDXL1 and GR-CDXL4 CDX and cell line; GR-CDXL3 TB, CDX, and cell line). Large-scale alterations including gain in 7q containing *CDK6* (patient L2 TB and GR-CDXL2 CDX, GR-CDXL3, and GR-CDXL4 CDX and cell line) were also found. In addition, *AKT1* gain was found in GR-CDXL3 CDX and cell line, as well as *ERBB2* (chr 17) amplification (Supplemental Figure 8). Comparing WES data with significantly altered genes in adenocarcinoma and squamous cell carcinoma from 11 cBioPortal studies in NSCLC showed that our models were highly representative of these histologies (Supplemental Figure 10, A and B). These variants were also predominant in other types of metastatic malignancies (Supplemental Figure 10C) (30). In addition, key DDR-related alterations that emerge from our CDX model WES analysis are found at a low frequency in cBioPortal NSCLC studies (31–35).

**Figure 5:**
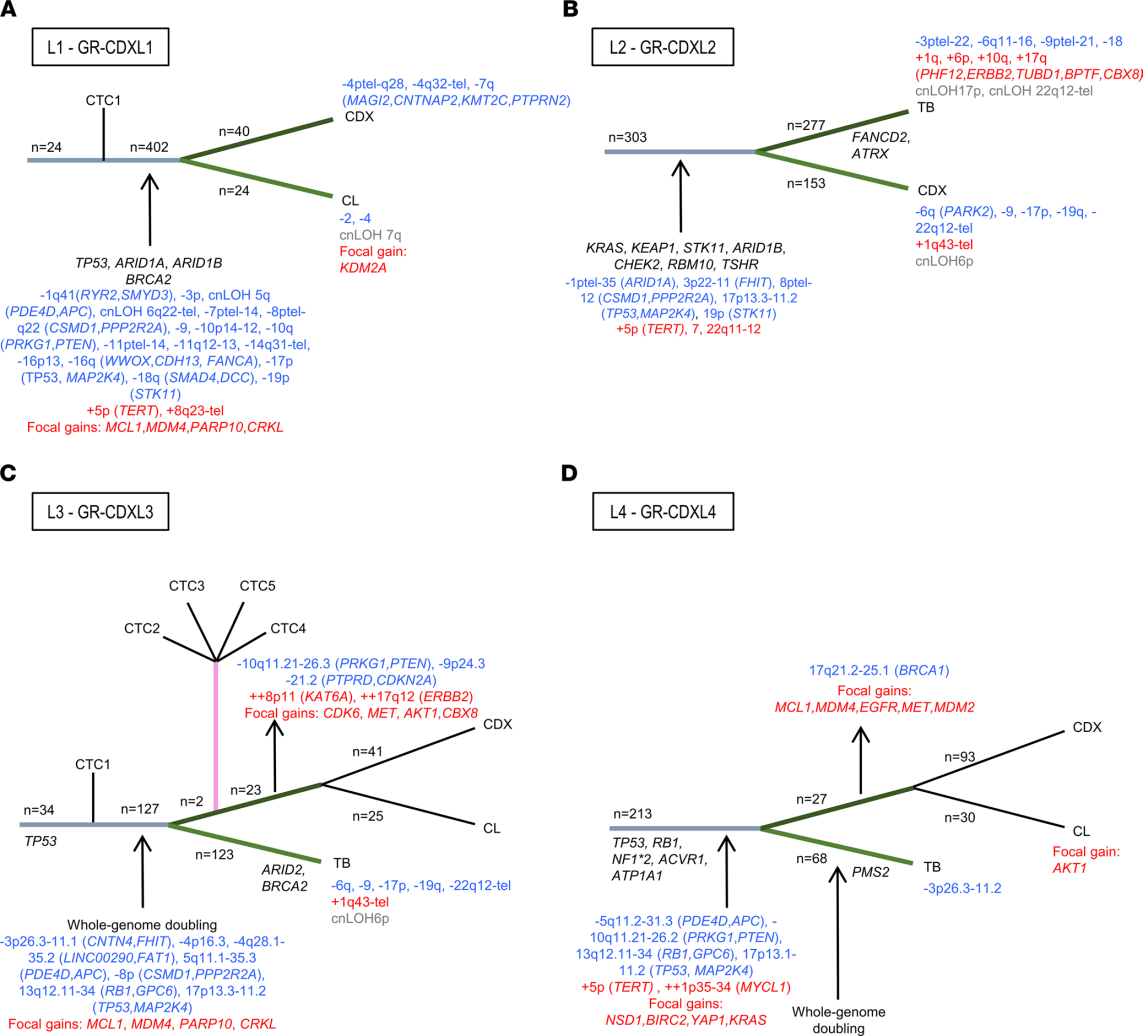
Phylogeny of CDX and CDX-derived cell lines. (**A**–**D**) Branches are unscaled, and their length is not proportional to the number of alterations occurring in the branch. The number of mutations (in dark) and the CNAs (gain in red and loss in blue) are mentioned on the branches of the tree. Only genes bearing driver truncal or acquired alterations (mutations or CNAs) are indicated.

Finally, using driver alterations, we investigated cell lineage tracing of the CDX and CDX-derived cell lines. Phylogenetic inference from somatic mutations and CNA data was used to map the clones from TB and tumorigenic CTCs that contributed to CDX tumors (Figure 5). In the 4 CDX models, loss of tumor suppressor genes such as *TP53*, *STK11*, and *MAP2K4* was an early event. Subclonal mutations in *TP53*, *ARID1B*, and *BRCA2* genes were detected in GR-CDXL1 CDX and cell line (Figure 5A). Subclonal loss of the 16q region harboring DNA repair–related *WWOX* and *FANCA* was also observed in this model (Figure 5A). Truncal cooccuring mutations were found in oncogenic driver genes *KRAS*, *KEAP1*, *STK11*, *ARID1B*, *RBM10*, and *TSHR* in patient L2 TB and GR-CDXL2 CDX (Figure 5B). Moreover, truncal driver alterations were detected in DDR pathway genes *CHEK2* and *ARID1B* (Figure 5B). In GR-CDXL3, truncal *TP53* mutation and early whole-genome doubling (WGD) were detected, as were clonal *MDM4* and *PARP10* gains (Figure 5C). Two ramifications were observed: the first one containing patient L3 single CTCs 2-5, the CDX, and the cell line — which acquired *PTEN* loss, *BRCA1* loss, *ERBB2* amplification, and *AKT1* gain — and the second one carrying the TB (Figure 5C). In GR-CDXL4, *TP53*, *RB1*, *NF1*, *ACVR1*, and *ATP1A1* driver mutations were clonal, while gains of DNA repair–related genes *MDM2* and *MDM4* were subclonal in the CDX and the cell line (Figure 5D). Overall, phylogenetic reconstruction of the 4 models reveals the clonality of mutations in DDR- and repair-related genes, suggesting their potential implication in metastatic disease progression and interest as therapeutic targets. CNAs in key DNA repair genes are also acquired in CDX and cell lines, recapitulating important CIN in our models.

### DDR and DNA repair mechanisms activity in CDX-derived cell lines.

**Figure 6:**
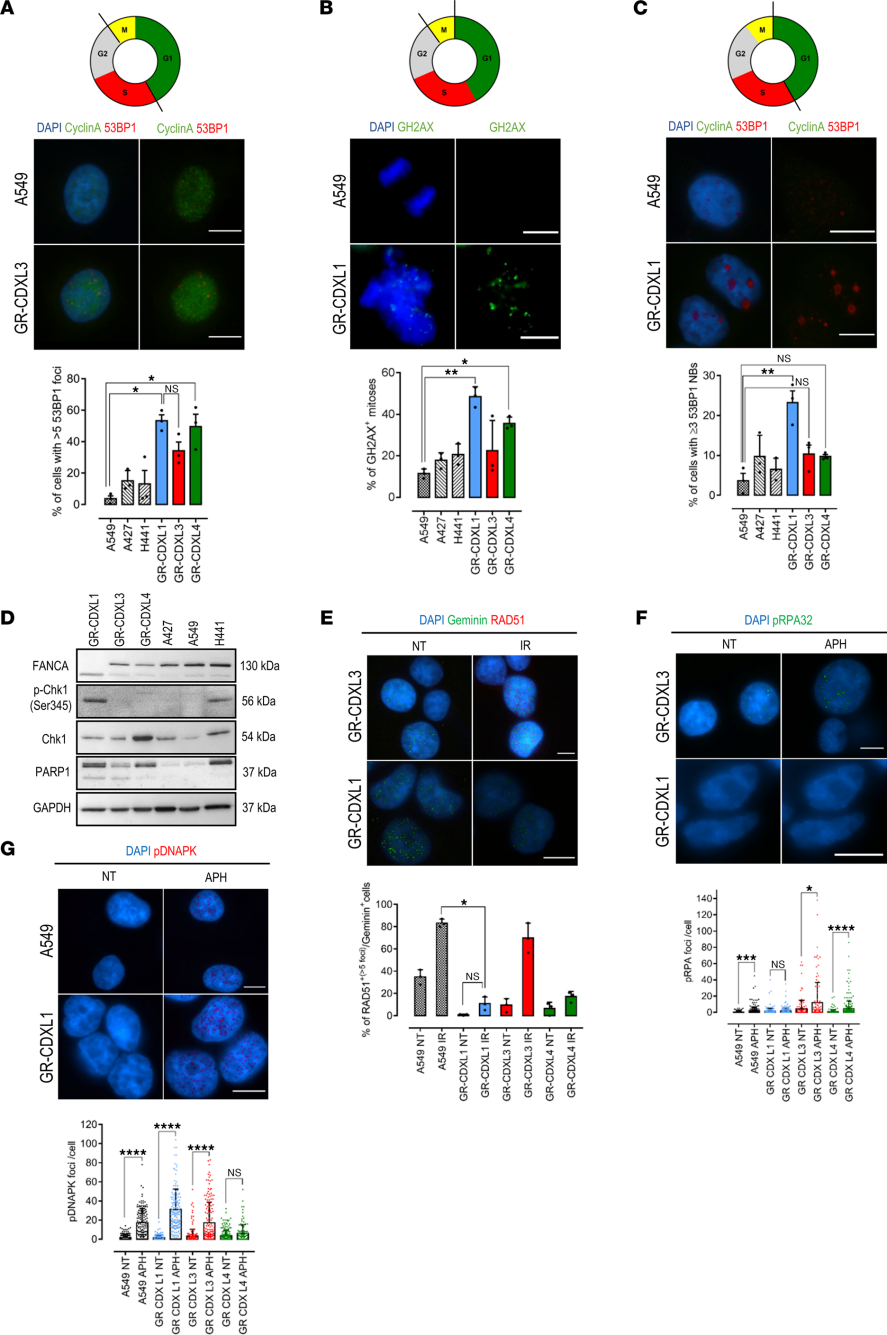
DNA damage response activation in CDX-derived cell lines. (**A**) Representative images of 53BP1 foci (red) in A549 and GR-CDXL3 cells (top). Proportion of S/G2 (cyclinA^+^) cells with more than 5 53BP1 foci (bottom). (**B**) Representative images of GH2AX^+^ (green) mitotic cells in GR-CDXL1 and A549 (top). Proportion of H2AX^+^ mitoses (bottom). (**C**) Representative images of 53BP1 NB (red) in A549 and GR-CDXL1 cells (top). Proportion of G1 (cyclinA^–^) cells with more than 3 53BP1 NB (bottom). (**D**) Western blot analysis of the levels of p-CHK1, CHK1, FANCA, and PARP1 in CDX-derived and NSCLC cell lines. (**E**) Representative images of IR-induced RAD51 foci (red) in S phase (geminin^+^) GR-CDXL1 and GR-CDXL3 cells (top). Proportion of RAD51^+^/geminin^+^ NT and IR cells (bottom). (**F**) Representative images of APH-induced pRPA32 foci (green) in GR-CDXL1 and GR-CDXL3 cells (top). Level of pRPA32 foci per cell in NT and APH-treated (bottom). (**G**) Representative images of APH-induced pDNA-PK foci (red) in A549 and GR-CDXL1 cells (top). Level of pDNAPK foci per cell in NT and APH-treated (bottom). For **E**–**G**, we kept only A549 NSCLC cell line as a comparator, as the other control had equivalent levels of DNA damage. Data are shown as mean ± SD from at least 3 independent experiments; *P ˂ 0.05, **P ˂ 0.01, ***P ˂ 0.001, ****P < 0.0001 by Kruskall Wallis and post hoc Dunn’s test. NB, nuclear body; NT, nontreated, IR, irradiated; APH, aphidicolin. Scale bar: 10 μm.

The recurrence of DDR-related genomic alterations revealed by WES led us to the mechanistic characterization of DDR mechanisms in our models. To estimate DSB frequency in CDX-derived cell lines, a dual cyclin A (S/G2 phase marker)/p53-binding protein 1 (53BP1-mediator of DSB repair) staining was performed. 53BP1 localizes to lesions and forms foci during S/G2 phases (Figure 6A). The incidence of DSBs in S/G2 is significantly higher in CDX-derived cells (ranging from 38%–58% of cells) compared with control NSCLC adenocarcinoma cell lines (Figure 6A). Phosphorylation of histone H2AX was also assessed to monitor DNA damage in cancer cells undergoing mitosis. We observed important levels of damaged mitotic DNA in GR-CDXL1 and in GR-CDXL4 compared with control (Figure 6B). Notably, GR-CDXL1 G1 cells harbored a significant proportion of 53BP1 nuclear bodies, which indicates the persistence of unrepaired damage after mitosis in G1 (Figure 6C). Furthermore, persistent DNA damage in GR-CDXL1 induced constitutive activation of checkpoint kinase 1 (*CHEK1*) shown by increased phosphorylation at Ser-345 distinctly in GR-CDXL1 cells (Figure 6D).

Next, we monitored nuclear foci of key actors in HR and nonhomologous end joining (NHEJ) to evaluate DSB repair in the CDX-derived cell lines. HR activity was assessed through RAD51 and phosphorylated RPA32 (pRPA) recruitment. RAD51 foci were detected in geminin-expressing S-phase cells after induction of DSBs by ionizing radiation (IR). While HR was activated upon IR in ~80% of GR-CDXL3 cells, RAD51 recruitment was negligible in GR-CDXL1 (10% of cells) and GR-CDXL4 (~20%) (Figure 6E). Similarly, pRPA32 failed to be recruited at lesion sites in GR-CDXL1 cells after aphidicolin (APH; replicative DNA polymerase inhibitor) treatment (Figure 6F). On the other hand, GR-CDXL1, GR-CDXL3, and GR-CDXL4 cells were NHEJ proficient in response to APH-induced DNA damage, as shown by pDNA-PKc nuclear foci formation (Figure 6G). These results reveal HRD in GR-CDXL1 cells, which is concordant with somatic *BRCA2* mutation and *FANCA* loss detected by WES (Figure 5A and Supplemental Figure 6). HRD was also observed in GR-CDXL4, without any genomic rationale behind it. Interestingly, high PARP1 protein levels were detected in GR-CDXL1 and GR-CDXL4 cells, suggesting its possible targeting (Figure 6D).

### Mitotic defects investigation in CDX-derived cell lines.

**Figure 7:**
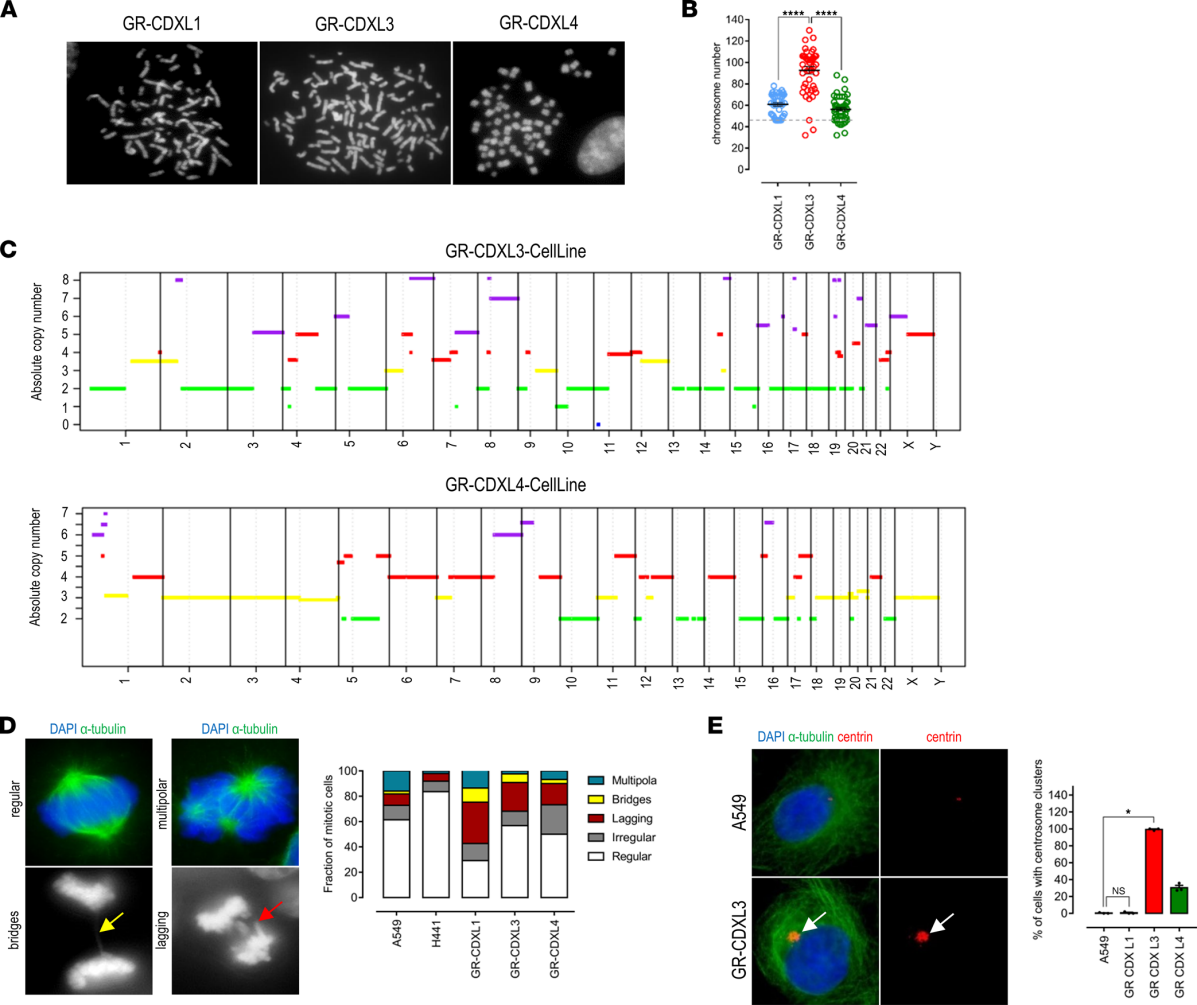
Mitotic defects in CDX-derived cell lines. (**A**) Metaphase spreads of GR-CDXL1, GR-CDXL3, and GR-CDXL4 chromosomes, shown at a total magnification of ×150. (**B**) Chromosome numbers. (**C**) Absolute copy number profiles showing whole-genome duplication of GR-CDXL3 (top) and GR-CDXL4 (bottom) cell lines. (**D**) IF analyses of mitotic defects in NSCLC and CDX-derived cells; yellow arrow indicates anaphasic bridge, and red arrow indicates lagging chromosome (left). Fraction of mitotic cells presenting defects (right). (**E**) Representative images of dual α-tubulin/centrin immunostaining revealing clustering of extra centrosomes in GR-CDXL3 cells (white arrows) (left). Proportion of cells presenting centrosome clusters (right). Statistical significance was assessed using Kruskall Wallis and post hoc Dunn’s test for **B** and **E**. Data are shown as mean ± SEM; *P ˂ 0.05, ***P < 0.0001 from *n* = 3 experiments.

High CIN level has been confirmed by metaphase chromosome spreads of GR-CDXL1, GR-CDXL3, and GR-CDXL4 cells, presenting 54, 110, and 59 chromosomes, respectively (Figure 7, A and B). A tetraploid DNA content was detected by WES in the GR-CDXL3 cell line only, while GR-CDXL4 exhibited a near-triploid genome (Figure 7C), recapitulating the copy number profiles of the corresponding CDX (Supplemental Figure 8 and Supplemental Figure 12A). These results, supported by the presence of centromeres into lagging DNA during mitosis (Supplemental Figure 12, B and C), highlight numerical CIN distinctly in GR-CDXL3. Furthermore, CDX-derived cells presented numerous mitotic defects including multipolar divisions, anaphase bridges and lagging chromosomes (Figure 7E). We, thus, focused next on centrosome abnormalities, which are frequent in cancer and contribute to CIN (36). Clustering of supernumerary centrosomes has been previously reported as a coping mechanism of cancer cells, enabling them to form bipolar spindles and survive (37). To investigate whether this event is a common feature in our models, dual α-tubulin/centrin IF staining was performed (Figure 7E). Interestingly, GR-CDXL3 exhibited high proportions of cells with centrosome clustering (~85%) compared with control A549, GR-CDXL1 and GR-CDXL4 cells. This phenomenon is known to protect cancer cells from otherwise lethal multipolar divisions, which is consistent with their low incidence level in GR-CDXL3 cells (Figure 7, D and E).

### In vitro, in ovo, and in vivo therapeutic targeting of CDX-derived cell lines.

**Figure 8:**
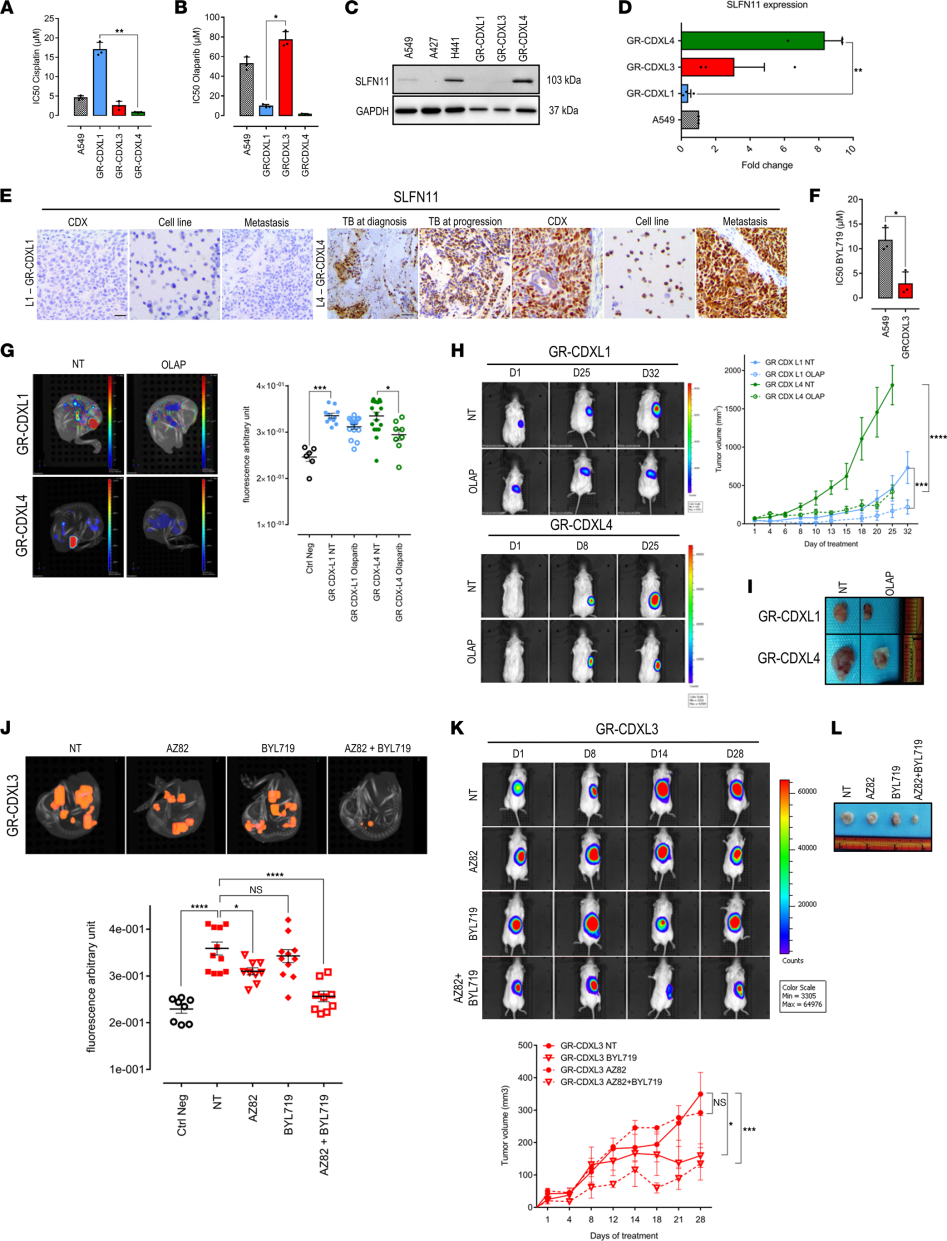
In vitro, in ovo, and in vivo drug assays. (**A**) Mean in vitro IC_50_ values of cisplatin for control and CDX-derived cell lines. (**B**) Mean in vitro IC_50_ values of olaparib. (**C**) Western blot showing SLFN11 expression levels in GR-CDXL1, GR-CDXL3, GR-CDXL4, and NSCLC cell lines. (**D**) qPCR for SLFN11 gene expression in A549 and CDX-derived cell lines normalized to GAPDH expression level. Data are fold change and are shown as mean ± SEM. *n* = 3 experiments; **P ˂ 0.01 by Kruskall-Wallis and Dunn’s test. (**E**) IHC staining of SLFN11 in patients L1 (CDX, cell line, and metastatic mouse tumor) and L4 (TB, CDX, cell line, metastatic mouse tumor) samples. Scale bar: 30 μm. (**F**) Mean in vitro IC_50_ values of BYL719 for control and GR-CDXL3 cell line. For **A**, **B**, and **F**, data are shown as mean ± SD. *n* = 3 experiments; *P < 0.05, **P < 0.01, Kruskall-Wallis and Dunn’s test (**A** and **B**), 2-tailed unpaired *t* test with Welch’s correction (**F**). (**G**) Three-dimensional representative images at ID17 (left) and quantitative analysis of average fluorescent tumor foci (right) of GR-CDXL1 or GR-CDXL4 mCherry-expressing CAM tumors, treated or not with olaparib. (**H**) Luciferase-expressing GR-CDXL1 (left, upper panel) or GR-CDXL4 (left, lower panel) tumors treated with olaparib. Representative BLI images (left) and tumor volumes (right) obtained at indicated days of treatment are shown. (**I**) Tumors at day 32 (GR-CDXL1-Luc) and day 25 (GR-CDXL4-Luc). (**J**) Three-dimensional representative images obtained at ID17 (left) and quantitative analysis of average fluorescent tumor foci (right) of GR-CDXL3 mCherry-expressing CAM tumors treated or not with AZ82 and/or BYL719. For **G** and **J**, data are shown as mean ± SEM. *n* = 3 experiments; *P ˂ 0.05, **P ˂ 0.01, ***P ˂ 0.001, ****P < 0.0001 by Kruskall-Wallis and Dunn’s test. Each point represents a single embryo. (**K**) Representative BLI images of GR-CDXL3 luciferase–expressing mouse tumors treated or not with AZ82 and/or BYL719. Tumor volume is shown (lower panel). For **H** and **K**, data are shown as mean ± SEM. *n* = 5; *P ˂ 0.05, ***P ˂ 0.001, ****P ˂ 0.0001 by 2-way ANOVA. (**L**) GR-CDXL3 luciferase–expressing tumors obtained at day 28.

WES-based mutation landscape and subsequent mechanistic studies highlighted CIN and genome instability propagation across all CDX models, suggesting cancer cell vulnerabilities. This, thus, provided a biological rationale for the selection of drug candidates targeting DNA repair and DDR defects (Supplemental Figure 13). First, GR-CDXL1 resistance to cisplatin and sensitivity in GR-CDXL4 observed in vivo were confirmed in vitro (Figure 8A). HRD in GR-CDXL1 and GR-CDXL4 cell lines (Figure 6, E and F) led us to assess the efficacy of PARPi olaparib. In spite of cisplatin resistance but in concordance with its HRD features, GR-CDXL1 responded to olaparib. GR-CDXL4 was also extremely sensitive to olaparib compared with A549 and GR-CDXL3 cells (Figure 8B). As no biological explanation was provided for drug response in GR-CDXL4, we explored a hypothesis based on a protein biomarker. We, therefore, assessed SLFN11 expression in our cell lines and its possible correlation with GR-CDXL4 cell sensitivity to olaparib. Interestingly, GR-CDXL4 cells overexpressed SLFN11 protein, while it was not detected in A549, GR-CDXL1, or GR-CDXL3 cells (Figure 8C). *SLFN11* mRNA levels were also significantly higher in GR-CDXL4 cells (8-fold) compared with other cell lines (Figure 8D). These findings led us to further investigate SLFN11 expression in olaparib-sensitive GR-CDXL1 and GR-CDXL4 samples. To this end, we performed IHC on GR-CDXL1 CDX and cell line, patient L4 TB specimens at diagnosis and progression, and GR-CDXL4 CDX and cell line. Representative samples from NSG mice metastases (see Figure 2B) from both CDX models were also analyzed. IHC analysis revealed that 60% of tumor cells in patient L4 TB (diagnosis and progression) were positive for SLFN11. GR-CDXL4 samples also strongly expressed SLFN11 with 70%, 90%, and 90% cell positivity in the CDX, the CDX-derived cell line, and the mouse metastatic tumor, respectively. In contrast, GR-CDXL1 tumor samples were all negative for this marker (Figure 8E). Together, these data indicate that SLFN11 overexpression may be implicated in reduced HR activity in GR-CDXL4 cells, conferring olaparib sensitivity independently of *BRCA1*/*2* mutation status.

In ovo*,* both GR-CDXL1 and GR-CDXL4 mCherry-expressing tumors responded to a 6-day course of olaparib monotherapy (100 μg/kg). Indeed, the metastatic fluorescence signals obtained by GR-CDXL1 and GR-CDXL4 tumors on the CAM were significantly reduced at ID17 (Figure 8G and Supplemental Figure 14B). In vivo*,* significantly delayed tumor growth was observed in treated groups as expected, while nontreated GR-CDXL1-Luc and GR-CDXL4-Luc tumors were unresponsive, reaching respectively twice and 4 times the initial tumor volume over the experimental course (Figure 8, H and I, and Supplemental Figure 14C).

A panel of DDR and cell cycle inhibitors including NHEJ key factor DNA-dependent protein kinase (DNAPK) inhibitor NU7441 and Aurora A inhibitor alisertib was evaluated in our CDX-derived cell lines. However, no significant effects have been noted (data not shown). GR-CDXL3 cells were highly sensitive to PI3KA inhibitor BYL719 in vitro compared with control, in accordance with *AKT* amplification and *PTEN* loss detected in CNA analysis (Figure 8F and Supplemental Figure 14A). We next tested whether centrosome clustering inhibition would impact GR-CDXL3 cell survival by targeting kinesin family member C1 (KIFC1), a critical factor in this mechanism. No significant effect of KIFC1 inhibitor AZ82 on GR-CDXL3 cells was found in vitro (data not shown). We then sought to compare GR-CDXL3 tumor response to BYL719 and AZ82 as monotherapy versus combination therapy in the CAM (Figure 8J). The effect of BYL719 was not statistically significant, while tumors were slightly more responsive to AZ82 alone. Interestingly, a notable synergistic effect of the drug combination was observed on GR-CDXL3 tumors compared with monotherapy (Figure 8J). In contrast to the in ovo assay, AZ82 monotherapy did not have a noticeable effect on tumor growth in NSG mice, while tumor response to BYL719 was statistically significant (Figure 8K). Most importantly, tumors were highly sensitive to the AZ82/BYL719 combination targeting 2 different mechanisms of tumor adaptation in GR-CDXL3, in concordance with the synergy obtained in ovo (Figure 8, K and L, and Supplemental Figure 14, D and E).

## Discussion

Defective DDR and genome instability are common in NSCLC and a potential therapeutic opportunity, but clinical data have so far been disappointing (14). In this study, we report the comprehensive analysis of 4 CDX models established from advanced-stage NSCLC patient CTCs — which recapitulated patient tumor pathology and chemoresponse — and 3 CDX-derived cell lines. Genomic analysis by WES unraveled a characteristic mutational spectrum from which several DNA repair-related deleterious alterations emerged, associated with CTC-mediated metastatic progression. Mechanistic studies revealed high levels of DNA damage in our CDX-derived cell lines, notably in GR-CDXL1 where DSBs remain unrepaired after mitosis. Subsequent functional assessment evaluation of DNA repair activity showed impaired RAD51 foci formation in GR-CDXL1 and GR-CDXL4 cells, accompanied by sensitivity to PARPi in vitro, in the CAM model and immunodeficient mice, thus inferring HRD. This was supported by a genomic rationale in GR-CDXL1 involving *BRCA2* mutation and *FANCA* deletion, while *SLFN11* overexpression was elucidated as a molecular rationale predictive of olaparib sensitivity in GR-CDXL4. GR-CDXL3 presented supernumerary chromosomes and centrosome clustering, leading to CIN propagation and promoting highly aggressive tumor seeding in ovo and in vivo. Moreover, GR-CDXL3 tumors were highly sensitive to the drug combination targeting centrosome clustering and AKT signaling.

One fundamental limitation is the low prevalence of CellSearch-detected epithelial CTCs in some metastatic cancers (e.g., NSCLC, pancreatic cancer). Only 1 CDX model has been previously generated in NSCLC, displaying a predominant mesenchymal phenotype (22). Here, we report the comprehensive analysis of 4 CDX models established from advanced-stage NSCLC patient EpCAM^+^ CTCs and 3 CDX-derived cell lines. CTC counts as low as 35 were sufficient for CDX tumor growth, indicating that, even at low concentrations, CTCs may contain subpopulations with important tumorigenic potential. Overall engraftment rate was low (4 of 55, ~7%) as expected. We observed that it was higher for squamous cell carcinoma (1 of 7 patients; 14.3%) than adenocarcinoma (3 of 45 patients; 6.67%). However, it is difficult to draw a correlation between success rates and tumor histologies based on such a low number of successful attempts. In our study, all CDX tumors and established cell lines presented an epithelial phenotype, which shows that EpCAM^+^ CTCs presented tumorigenic potential in our 4 models. In vivo drug assays recapitulated patient responses to first-line chemotherapy, thus validating our models. CDX-derived cell lines have demonstrated strong tumorigenic activity both in ovo and in vivo. Notably, GR-CDXL3 and GR-CDXL4 cells were highly metastatic, in concordance with their CIN profiles, showing their relevance as a tool to investigate mechanisms underlying metastatic progression, as was previously reported using small cell lung cancer (SCLC) CDX-derived cells (38). WES analysis showed that GR-CDXL2 and GR-CDXL3 CDX recapitulated the corresponding patient diagnostic TB, while GR-CDXL4 was representative of the TB at progression, with 52%, 57%, and 76 % mutational profile similarity. Moreover, 34%, 29%, and 36% of mutations were found exclusively in the GR-CDXL2, GR-CDXL3, and GR-CDXL4 CDX, respectively, possibly issued from metastasis. The patient L1 biopsy specimen was insufficient for molecular profiling, as biopsy material is often scarce in NSCLC. The relevance of GR-CDXL1, thus, relies on the molecular similarity with patient L1 CTC1 represented by 24 shared mutations. In accordance with NSCLC cBioPortal studies, we found that genes harboring truncal mutations in GR-CDXL1, GR-CDXL2, and GR-CDXL4 were found in 79% of genes altered in lung adenocarcinoma, while truncal mutations in GR-CDXL3 are found in 61% of genes altered in squamous cell carcinoma. Therefore, our models were found to be representative of NSCLC histologies, displaying alterations exclusive to this malignancy (e.g., *TP53*, *KRAS*, *KEAP1*, *STK11*). Key DDR-related mutations emerge from WES analysis, including *TP53*, *BRCA2* (GR-CDXL1), *CHEK2* (GR-CDXL2), and *ARID1B* (GR-CDXL1, GR-CDXL2), and reconstruction of phylogenetic trees infers their clonality. In addition, important subclonal CNA diversification in DDR-related genes was revealed across our models. Overall, genomic analysis supports the hypothesis that defects in genome maintenance mechanisms fuel CTC-driven tumor progression in NSCLC models.

Based on the rationale that DNA repair impairment may sensitize tumor cells to DNA-damaging chemotherapy, several clinical trials were conducted to assess PARPi efficacy in combination with chemotherapy in NSCLC patients (11). Despite encouraging phase II results in metastatic squamous NSCLC, phase III evaluation of veliparib in association with chemotherapy failed to show any survival benefits (34, 35). In the maintenance setting, PIN and PIPSeN trials have shown that olaparib also failed to improve survival in platinum-sensitive NSCLC patients (39, 40). However, patients were not included based on HRD status. Others are currently evaluating PARPi activity with or without chemotherapy in NSCLC patients, harboring HRD or not (12). In a recent study assessing the occurrence of HR-related mutations across several cancers, HRD was reported in 5% of NSCLC patients and 2% of *BRCA1/2* variants were pathogenic in this population (41). Here, we report that GR-CDXL1 displays a somatic biallelic mutation in *BRCA2* and promoter deletion of Fanconi anemia pathway gene *FANCA*. The 2 pathways are in crosstalk for DNA lesion repair, and *BRCA2* and *FANCA* inactivation promote HRD, which is also evident through unrepaired damage after mitosis, constitutive activation of *CHEK1*, and failure in RAD51 foci formation in GR-CDXL1 cells. Interestingly, similarly to patient L1, the GR-CDXL1 model is resistant to chemotherapy but, as predicted by molecular and functional profiling, highly sensitive to olaparib in vitro, in ovo, and in mice. This previously undescribed clinical context suggests that resistance to chemotherapy does not exclude PARPi efficacy in HR-deficient NSCLC tumors. A deeper understanding of the biological basis of HRD is, thus, crucial to expand patient screening beyond chemosensitivity for a more adequate selection of patients with HRD features and optimize PARPi efficacy in NSCLC malignancies.

We report a second HR-deficient CDX model GR-CDXL4, sensitive to olaparib but lacking an HRD-related mutation. SLFN11, an acknowledged DNA/RNA helicase recruited at replication forks via replication protein A (RPA) in response to genotoxic stress, has recently emerged as a candidate biomarker of sensitivity to platinum-based chemotherapies and PARPi (39–41). In SCLC, SLFN11 expression correlated with PARPi olaparib and talazoparib activity in preclinical models, while it was associated with improved survival in patients treated with PARPi/chemotherapy combination (28, 42, 43). To our knowledge, its significance in NSCLC has not been reported yet. Interestingly, in this study, we detected high *SLFN11* mRNA levels and SLFN11 protein overexpression in GR-CDXL4 cells. IHC analysis indicated a strong expression of SLFN11 in patient L4 TB at progression and in the corresponding CDX tumor. This prompted us to evaluate SLFN11 expression in patient L4 TB at diagnosis, which revealed strong expression, as well. Basal SLFN11 expression may, thus, be predictive of tumor sensitivity to PARPi. Nevertheless, additional investigations are warranted to confirm the correlation between SLFN11 overexpression and NSCLC tumor response to olaparib. Initially, the patient L4 TB specimen at diagnosis was classified as *MET*-amplified adenocarcinoma, and additional analysis showed positive neuroendocrine staining in a few tumor cells, in addition to *SLFN11*^+^ cells. At progression, neuroendocrine marker levels were intensified along with strong SLFN11 expression, suggesting a potential SCLC transformation. Histological transformation of lung adenocarcinoma into SCLC is a rare event, which has been described as a key resistance mechanism to tyrosine kinase inhibitor (TKI) treatment in around 5% *EGFR*-mutant and few *ALK*-rearranged NSCLC cases (44, 45). Furthermore, phylogenetic analysis by Lee et al. suggested that early divergent evolution of EGFR TKI-resistant SCLC from adenocarcinoma is predisposed by the complete inactivation of *RB1* and *TP53* (46). In the present case, we showed that all L4 tumor samples harbored loss-of-function *RB1* and *TP53* mutations. We propose that early tumor screening for SLFN11 expression can aid in the selection of NSCLC patients eligible for PARPi treatment. In addition to its predictive value of sensitivity, our data raise the hypothesis that SLFN11 expression may be a histologic biomarker to predict phenotypic transformation of adenocarcinoma into SCLC. Further investigation is needed to confirm this predictive role of SLFN11 in NSCLC malignancy.

WGD was observed as a clonal event in GR-CDXL3 model, consistent with previous work in NSCLC (47). The cell line displayed important CIN and was, thus, highly tumorigenic and seeded multiple metastases when injected in mice and chick embryo CAM. This ensued notably due to clustering of supernumerary centrosomes mediated by KIFC1, promoting bipolar divisions in cancer cells allowing their survival (48, 49). Since KIFC1 inhibition alone had no significant anticancer activity, we sought to improve its efficacy by adding PI3KA inhibitor BYL719, targeting AKT1 in GR-CDXL3 tumors. Synergy was observed between the 2 molecules, showing significant tumor response in ovo and in vivo. However, we acknowledge limitations to these data, as the AZ82 molecule has been shown to have unfavorable cytotoxic effects and none of the other KIFC1 inhibitors currently available exhibit enough potency (50). Nevertheless, encouraging findings recently reported by Fan et al. elucidated a potential role of KIFC1 as a biomarker of cancer recurrence. The authors show that DNA-damaging therapies promote centrosome clustering through ATM and Rad3-related–mediated (ATR-mediated) and Ataxia-Telangiectasia mutated–mediated (ATM-mediated) phosphorylation of KIFC1, suggesting this mechanism as a therapeutic target (50).

In conclusion, in-depth characterization and functional analysis of CDX systems elucidate a biological rationale for DDR and genome instability–directed therapeutics in NSCLC (Supplemental Figure 13). All our CDX models display a characteristic mutation spectrum for genome integrity regulator genes, highlighting their implication in CTC tumorigenic potential. Mechanistic studies unravel CTC-specific DNA repair dysfunctionality in 3 CDX and their corresponding cell line, which provides insight into the importance of DNA repair management in NSCLC. Importantly, our findings shed light on the necessity to broaden screening approaches in NSCLC beyond chemosensitivity in order to expand the category of patients who may benefit from PARPi. We suggest *SLFN11* as a predictive biomarker of sensitivity to PARPi in NSCLC. Additionally, we put forward its potential role as a predictor of NSCLC adenocarcinoma transformation into SCLC.

## Methods

Supplemental Methods are available online with this article.

### Patient samples

Blood was drawn in CellSave (Menarini Silicon Biosystems) and EDTA tubes and were immediately transferred to the laboratory.

### CTC enumeration

Blood samples collected in CellSave tubes were run with the CellSearch platform (Menarini) using the CTC kit (Menarini) according to manufacturer’s instructions and training.

### CTC enrichment before implantation into mice

In total, 50 μL of the RosetteSep cocktail (StemCell Technologies) was added per 1mL of blood and incubated 20 minutes at room temperature (RT). After incubation, the sample was diluted with an equal volume of HBSS (Invitrogen) supplemented with 2% FBS (Invitrogen). The solution was then carefully layered on top of 15 mL Ficoll-Plaque Plus (GE-Healthcare) and centrifuged for 20 minutes at 1200*g* at 20°C without brake. Enriched cells were collected, washed with 50 mL HBSS/2% FBS, and centrifuged for 5 minutes at 250*g* at 20°C. Cells were resuspended in 100 μL of cold HBSS supplemented with 100 μL cold Matrigel (Corning) and kept on ice until implantation in mice.

### Growth of CDX in immunocompromised mice

Before CTC implantation, NSG 6-week-old male mice (Charles River Laboratories) were anesthetized by peritoneal injection of 10 mg/mL ketamine and 1 mg/mL xylazine at a dose of 10 mL/kg. The upper dorsal regions of mice were shorn, and the skin was aseptized with a chlorhexidine solution, incised at the level of the interscapular region, and CTCs were injected in 200 μL HBSS/Matrigel in the interscapular fat pad. Mice were monitored every day. Palpable tumors were monitored once a week, and tumor volume was determined as the following: (tumor length × tumor width^2^)/2. When it reached 1770 mm^3^ or when mice presented signs of deteriorated health status, tumors were aseptically excised and dissected into fragments of approximately 20 mm^3^. Tumor fragments were passaged into NSG mice, and the remainder of the tumor was used for Alu sequence detection, IHC, molecular analysis, and cell line establishment. Mice were housed in pathogen-free animal housing at the Center for Exploration and Experimental Functional Research (CERFE; Evry, France) animal facility in individually ventilated cages of Polysulfone plastic (213 × 362 × 185 mm) with sterilized and dust-free bedding cobs; they were provided access to sterilized food and water ad libitum, under a light-dark cycle (14-hour circadian cycle of artificial light) and with controlled RT and humidity. Mice were housed in groups with a maximum of 6 animals during a 7-day acclimation period and groups of a maximum of 6 animals during the experimental phase.

### Enrichment, detection and isolation of single CTCs

Individual CTC isolation was performed by combining different methods described in Supplemental Methods.

### WES

Genomic DNA is captured using Agilent in-solution enrichment methodology (SureSelect XT Clinical Research Exome, Agilent) with their biotinylated oligonucleotides probes library (SureSelect XT Clinical Research Exome 54 Mb, Agilent), followed by paired-end 75 bases massively parallel sequencing on Illumina HiSeq4000. For the detailed process, see Gnirke et al. (51). Sequence capture, enrichment and elution are performed according to manufacturer’s instruction and protocols (SureSelect, Agilent) without modification except for library preparation performed with NEBNext Ultra kit (New England Biolabs). For library preparation, 600 ng of each genomic DNA are fragmented by sonication and purified to yield fragments of 150–200 bp. Paired-end adaptor oligonucleotides from the NEB kit are ligated on repaired, A-tailed fragments and then purified and enriched by 8 PCR cycles. In total, 1200 ng of these purified libraries are then hybridized to the SureSelect oligo probe capture library for 72 hours. After hybridization, washing, and elution, the eluted fraction is PCR amplified with 9 cycles; it is then purified and quantified by quantitative PCR (qPCR) to obtain sufficient DNA template for downstream applications. Each eluted-enriched DNA sample is then loaded on an Illumina HiSeq4000 for 75b paired-end sequencing. Image analysis and base calling were performed using Illumina Real Time Analysis (2.7.6) with default parameters.

### Heatmap

CNA absolute profiles were clustered using the inverse Spearman correlation coefficient as distance, and Ward’s aggregation method. The heatmap was built using the complexHeatmap R package and in-house codes. All computation and figure processings were performed using R v4.0.2.

### Phylogenetic inference

All nonsilent somatic mutations present in at least 2 tumor samples were considered for determining phylogenetic trees. Tree construction is detailed in Supplemental Methods.

### CDX-derived cell line establishment and cell culture

CDX tumor dissociation and cultures are described in Supplemental Methods.

### Immunofluorescence

Cells were seeded on coverslips in 6-well plates. After 48 hours, cells were either left untreated, irradiated (6 Gray) or treated with APH at the indicated doses. Twenty-four hours later, cells were washed with PBS 1×, fixed with 4% paraformaldehyde (15 minutes at RT), and permeabilized with 0.5 Triton X-100 (Roche; 10 minutes at RT). Nonspecific binding sites were blocked with BSA for 30 minutes. Primary antibody staining (Supplemental Table 7) was performed at 37°C for 1 hour, followed by a secondary antibody (anti–rabbit Alexa Fluor 555 [clone Poly4064, 406412, BioLegend]; anti–mouse Alexa Fluor 488 [polyclonal, A-11029, Invitrogen]) incubation of 30 minutes at 37°C. Scanning and image analysis were done on an Ariol scanning system (Leica Biosystems Richmond Inc.) including a Leica DM6000 B microscope with multibay stages (MB 8).

### RNA extraction and qPCR analysis

For *SLFN11* gene expression analysis, total RNA was isolated, and qPCR was performed as described in Supplemental Methods.

### In vitro drug assays

CDX-derived and A549 cells were seeded in quadruplicates into 384-well plates. Twenty four or 48 hours after seeding, cells were treated with cisplatin, olaparib, BYL719, or AZ82 for 5 days. Drugs were diluted in Advanced DMEM (Invitrogen). Cell viability assays were performed using CellTiter-Glo Luminescent Cell Viability assay kit (Promega). Luminescence was measured by Victor X4 Series Multilabel Plate Readers (Perkin Elmer). Generation of drug-response curves and determination of IC_50_ values were performed using Prism software.

### In vivo modeling of metastasis and drug assays

#### CAM.

Fertilized chicken eggs were purchased (EARL Les Bruyères) and incubated for 3 days at 37°C with 60% humidity. Metastasis evaluation and drug assays in the CAM chick embryo model were performed as previously described (29). Briefly, 2 × 10^3^ mCherry-expressing cells were implanted into the CAM at incubation day 10 (ID10). Tumor growth and embryo viability were examined daily until imaging analysis at ID17. Treatments were administered topically starting at ID11 as follows: olaparib via single administration (100 μg/kg), and AZ82 (20 μg/kg) and BYL719 (10 μg/kg) every other day. The final doses were calculated based on the weight of the chicken egg at ID11. At ID17, fluorescence and CT scans of the chick embryo were performed simultaneously using the IVIS Spectrum Imager (PerkinElmer). A 3D reconstitution of images was performed using Living Image software (PerkinElmer).

#### Mice.

In total, 5 × 10^5^ luciferase-expressing CDX-derived cells were grafted by IC injection into NSG mice (Charles River Laboratories). Metastatic progression was monitored once a week by bioluminescence imaging (BLI) evaluation under anesthesia and after i.p. injection of D-luciferin (15 μg/kg; Promega) using IVIS Spectrum imaging (PerkinElmer). To test in vivo drug efficacy, 2 × 10^6^ cells were injected s.c. into NSG mice. Once tumors reached an average volume of 100 mm^3^, mice were randomized into groups and treated with vehicle DMSO (1%), olaparib (50 mg/kg, i.p. injection, 3 times per week), BYL719 (20 mg/kg, oral gavage, 3 times per week), AZ82 (10 mg/kg, oral gavage, 3 times per week), or a combination of BYL719 and AZ82. S.c. tumor dimensions were measured by caliper. For BLI evaluation, i.p. with D-luciferin (15 μg/kg; Promega) was performed, and mice were scanned using IVIS Spectrum imaging (PerkinElmer) under anesthesia.

### Statistics

Unpaired 2-tailed Student’s *t* test with Welch’s correction was used to compare 2 groups, and Kruskall-Wallis followed by post hoc Dunn’s test was used for multiple comparisons. Two-way ANOVA was used to analyze tumor growth data in vivo. All statistical tests were performed using GraphPad Prism 7 software and are specified in figure legends. Data are shown as mean values ± SD or SEM, as indicated; 95% CI was used, and significance was considered when *P* value was less than 0.05.

### Study approval

#### Human studies.

The study (IDRCB2008-A00585-50) was conducted at Gustave Roussy, authorized by the French national regulation agency Agence Nationale de Sécurité du Médicament et des produits de santé (ANSM), and approved by the Ethics Committee and our IRB (CSET number 2008/1370). All patients provided written informed consent allowing for the collection of 10 blood samples (30 mL) over 3 years.

#### Animal studies.

Animal experimentation was approved by the Animal Experimentation Ethics Committee (no. 26, project 2018_019_13999) and performed according to European laws and regulations. The animal care, housing, and all experiments were performed in accordance with French legislation concerning the protection of laboratory animals and in accordance with a currently valid license for experiments on vertebrate animals, issued by the French Ministry of Higher Education, Research and Innovation (MESRI).

## Author contributions

TT, VF, and PP conducted experiments and contributed to experimental design, data analysis, and manuscript editing with the assistance of MO. AA provided technical assistance. OD, LBS, SC, and JGJ contributed to CDX establishment and characterization. PLK and PP provided DNA repair expertise. JR and SP contributed to data analysis and manuscript editing. JM, DP, and BB supported patient recruitment and sample management, and provided clinical support for the study. VM performed immunohistopathology experiments and analysis under the supervision of JYS. FF directed the research and manuscript editing.

## Supplementary Material

Supplemental data

Supplemental data set 1

Supplemental data set 2

Supplemental data set 3

Supplemental data set 4

Supplemental data set 5

Supplemental data set 6

Supplemental tables 1-7

## Figures and Tables

**Table 1:**
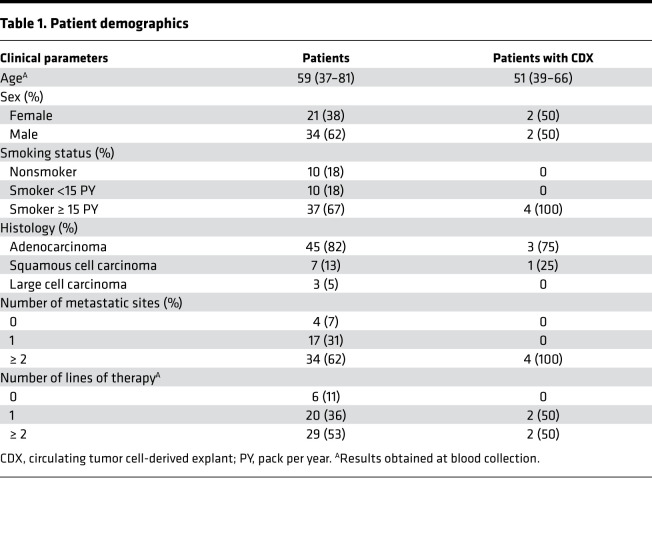
Patient demographics

## Version 1 05/05/2022

In-Press Preview

## Version 2 06/08/2022

Electronic publication
