# Supplementary material for: Targeting genome integrity dysfunctions impedes metastatic potency in non–small cell lung cancer circulating tumor cell–derived explants
Source: JCI Insight. 2022 Jun 8;7(11):e155804. doi: 10.1172/jci.insight.155804 (PMC9220846; doi:10.1172/jci.insight.155804)

SUPPLEMENTARY FIGURES

Supplementary Table 1: Clinicobiological characteristics of NSCLC cohort.

Supplementary Table 2: Number of variants identified in TB specimens, CDXs and CDX-derived cell lines.

Supplementary Table 3: Number of high-confidence variants identified in CTC samples.

Supplementary Table 4: List of antibodies used for IHC and staining conditions.

Supplementary Table 5: List of antibodies used for FACS analysis.

Supplementary Table 6: List of antibodies used for western blot.

Supplementary Table 7: List of antibodies used for immunofluorescence.

Supplementary Figure 1: Clinical timelines of patients L1, L2, L3 and L4. The length of each segment is proportional to the duration of treatment (1cm = 1 month). Patients L2, L3 and L4 biopsy at diagnosis was issued from the left latero-tracheal lymph node, the right inferior lobe and the left superior lobe of the lung respectively. Patient L4 biopsy at progression was sampled in the lung.

Supplementary Figure 2: Phenotypic characterization of patient samples, CDX and CDX-derived cell lines. (**A**) Number of CTCs detected by CellSearch according to tumor molecular status of the 55 NSCLC patients. (**B**) Representative images of cell morphology in the three CDX-derived cell lines at 10X, 20X and 40 X magnifications. Scale bar: 50 µm. (**C**) Immunohistochemical stainings on GR-CDXL1 CDX tumor and CDX-derived cell line (scale bar: 20 µm), patient L2 biopsy and GR-CDXL2 CDX tumor (scale bar: 30 µm) (HES, CK8/18, EpCAM, Ki67, Vimentin, TTF1,

Chromogranin A and synaptophysin) and on patient L3 biopsy, GR-CDXL3 CDX tumor and CDX-derived cell line (scale bar: 20 µm) (HES, chromogranin A, synaptophysin, P40, CK5/6, EpCAM, Vimentin, Ki67). All images are shown at 20X magnification. (**D**) Flow cytometry analyses of epithelial (EpCAM, panCK, E-cadherin), mesenchymal (vimentin) and cancer stem cell markers (ALDH, CD133, CD90, ABCG2 and CD166) in the three CDX-derived cell lines.

Supplementary Figure 3: In vivo drug assays. Palpable subcutaneous tumors of GR-CDXL1 (n=10), GR-CDXL2 (n=6), GR-CDXL3 (n=6) and GR-CDXL4 (n=9) at passages P11, P5, P5 and P4 respectively were treated once every week (intraperitoneal injection) with paclitaxel (15 mg/kg) and/or cisplatin (5 mg/kg) or the vehicle as indicated by the arrows. Tumor volumes were determined by caliper according to the formula $(width^2 \times length)/2$. Data are presented as mean tumor volumes ± SEM.

Supplementary Figure 4: Metastatic tumor detection in mice. GR-CDXL1, GR-CDXL3 and GR-CDXL4 tumor FFPE sections stained with HES, and anti-human CK7 and anti-human Ki67.

Supplementary Figure 5: Flow diagram of samples available for WES analysis for each patient.

Supplementary Figure 6: Comparative genomic analysis of the CDX and the CDX-derived cell lines. (**A**) Fraction of CDX mutations shared with the corresponding cell line. (**B**) Fraction of CDX driver mutations shared with the corresponding cell line. (**C**) Mutated driver genes and their amino acid sequence variation in the CDX and the CDX-derived cell line. (**D**) Mutated driver genes and their amino acid sequence variation in the CDX only.

Supplementary Figure 7: Dendograms resulting from hierarchical clustering analysis of all mutations detected by WES among patients L1, L3 and L4 samples.

Supplementary Figure 8: Comparative CNA analysis of the biopsies, the CDX and the CDX-derived cell lines. Circos plot of CNAs detected in the biopsy, the CDX and the CDX-derived cell line (when available) for each model (GR-CDXL1, GR-CDXL2, GR-CDXL3 and GR-CDXL4).

Supplementary Figure 9: Comparative CNA analysis of the CDX models. Circos plot of CNAs detected in the four CDX models (gain in red, normal in black, loss in blue) (1: GR-CDXL1, 2: GR-CDXL2, 3: GR-CDXL3, 4: GR-CDXL4).

Supplementary Figure 10: Frequency of CDX gene alterations in lung cancer histologies according to cBioPortal studies. (**A**) Genes harboring truncal mutations in GR-CDXL1, GR-CDXL2 and GR-CDXL4 were found in 79% of genes altered in lung adenocarcinoma. (**B**) Genes harboring truncal mutations in GR-CDXL3 are found in 61% of genes altered in squamous cell carcinoma. All CDX models displayed functional inactivation of *TP53* found at 53.8%. GR-CDXL1 harbored a deleterious *KMT2C* (9.4%), *ARID1A* (6.4%), *ATRX* (5.4%), *BRCA2* mutation (3.2%) and *FANCA* deletion (0.9%). GR-CDXL2 had a *KRAS*-mutant profile which included co-occurring mutations in *KEAP1* (14.5%), *STK11* (15.7%) and *RBM10* (9.5%) genes, exclusive of adenocarcinoma. Genomic characterization revealed DDR-related *CHEK2* and *ARID1B* driver mutations found at 1.4% and 3.4% respectively (1–5). (**C**) Genes harboring truncal mutations in GR-CDXL1, GR-CDXL2, GR-CDXL3 and GR-CDXL4 are predominant in other types of metastatic cancers.

Supplementary Figure 11: Statistics of allele drop-out and false-positive rate of single CTCs. (**A**) and (**B**) Representation of allelic drop-out (ADO) in GR-CDXL1 (**A.** left) and GR-CDXL3 (**B.** left). Reliable variants (green) were defined by an equal variant allele frequency (VAF) in both germline DNA and WBC bulk samples. Variants in ADO (red) were defined by a VAF ranging from 0.2 to 0.8 in germline DNA and <0.1 or >0.9 in WBC bulk. False-positive rates in one P8 CTC (**A.** right) and in five P51 CTCs (**B.** right) are shown. To estimate false-positive rates, we divided the number of reliable somatic variants not present in bulk tumor samples (TBs and CDX) by the number of target bases covered ≥8X in the same sample.

Supplementary Figure 12: Chromosomal instability of GR-CDXL3 and GR-CDXL4 CDX models. (**A**) Absolute copy number profiles of GR-CDXL3 and GR-CDXL4 CDX models. Yellow=normal/major copy number, red=gain, green=loss, purple=amplification. (**B**) Representative images of mitotic lagging chromosome analysis in GR-CDXL1 and GR-CDXL3 cells after 4’6-diamidino-2-phenylindole (DAPI, blue) and centromere staining with anti-CENPA (green). (**C**) Lagging DNA analysis obtained from scoring at least 50 anaphases per cell line in three independent experiments. Data are presented as mean ± SEM, *p<0.05 (unpaired t-test with Welch’s correction).

Supplementary Figure 13: Diagram of DDR-related pathways implicated in CDX-derived cell line vulnerabilities and targeting strategies. DNA damage in the three CDX-derived cell lines results in the activation of several repair cascades prone to defects. Olaparib-induced PARP trapping leads to synthetic lethality in *BRCA2*/*FANCA*-defective GR-CDXL1 cells. Dual targeting of AKT1 gain through the inhibition of PI3KA (by BYL719) and centrosome clustering via KIFC1 inhibition (by AZ82) leads to GR-CDXL3 cell death. In GR-CDXL4 cells, SLFN11 overexpression

may lead to olaparib sensitivity. SSB, single-strand DNA break; DSB, double-strand DNA break; HR, homologous recombination.

Supplementary Figure 14: *In vivo* parameters. (**A**) Western blot analysis of the levels of p-AKT and AKT in CDX-derived (GR-CDXL1, GR-CDXL3 and GR-CDXL4) and NSCLC (A427, A549 and H441) cell lines. (**B**) Average body weight of mice injected with GR-CDXL1 or GR-CDXL4 Luc cells measured over the course of treatment. (**C**) Quantitative analysis of BLI. (**D**) Average body weight of mice injected with GR-CDXL3 Luc cells measured over the course of treatment. (**E**) Quantitative analysis of BLI. Each point represents a single mouse. For **B-E**, n=5 for all groups except GR-CDXL1 NT where n=4. Data are presented as mean ± SEM, ***p<0.001, ****p<0.0001 (two-way ANOVA).

SUPPLEMENTARY METHODS

***Enrichment, detection and isolation of single CTCs*.** Individual CTC isolation was performed by combining different methods. Either: (i) isolation by size of epithelial tumor cells (ISET) filtration, immunofluorescence staining and scanning of filters, followed by laser microdissection CD45$^+$ cells, or (ii) enrichment via RosetteSep, immunofluorescence staining followed by FACS isolation, or (iii) CTC detection using CellSearch followed by CTC isolation using self-seeding microwell chips. The methods are described in detail in previous reports (6, 7).

***WGA, quality control, dsDNA*.** Whole genome amplification (WGA) of CTCs and CD45-positive cells was performed using the Ampli1 WGA kit (Menarini) according to manufacturer's instructions. The quality of Ampli1 WGA products was checked by multiplex PCR as described by Polzer *et al.* (8). To increase total dsDNA content in Ampli1 WGA products, ssDNA molecules were converted into dsDNA molecules using the Ampli1 ReAmp/ds kit (Menarini).

***Isolation of genomic DNA from blood, TB, CDX and CDX-derived cell line*.** DNA from formalin-fixed paraffin-embedded tumor biopsies was purified with QIAamp DNA FFPE Tissue kit (Qiagen, Hilden, Germany) according to manufacturer’s protocol. DNA from the CDX was extracted with AllPrep DNA/RNA kit (Qiagen) and germline DNA and cell line DNA was purified with QIAamp DNA blood kit (Qiagen).

***Sequence alignment and variant calling*.** Base calling was performed using the Real-Time Analysis software sequence pipeline (2.7.7) from Illumina with default parameters. Sequence reads from amplified DNA (circulating T cells and CD45 pools) were trimmed for Ampli1 adapters with Cutadapt (1.14) (9). Human reads from xenograft samples were extracted by bamcmp (10). Reads were then aligned to the

human genome build hg38/GRCh38.p7 using the Burrows-Wheeler Aligner (BWA) tool (11). Duplicated reads were removed using Sambamba (12). Variant calling of single nucleotide variants (SNVs) and small insertions/deletions (indels) was performed using the Broad Institute's GATK Haplotype Caller GVCF tool (3.7) (13, 14) for germline variants and MuTect2 tool (2.0, --max_alt_alleles_in_normal_count=2; --max_alt_allele_in_normal_fraction=0.04) (15) for somatic variants. To keep only reliable somatic variants, we then applied the following post-filtering steps:

- Variants classified as "PASS" or "t_lod_fstar" by MuTect2 (and not flagged as PID).
- Coverage ≥8 in the tumor and matched normal sample.
- QSS score ≥ 20.
- Variant allele fraction in the tumor (VAFT) ≥ 0.05 with ≥ 5 mutated reads, variant allele fraction in the normal sample (VAFN) = 0.

Additional criteria were applied to generate a high-confidence set of variants from CTCs. Variants had to be present in either the primary tissue (at least 1 TB specimen) or the CDX.

Bam-readcount (https://github.com/genome/bam-readcount) was used to rescue reliable variants that were present in at least two tumor samples and were not detected by Mutect2 because of their low VAF. Ensembl's Variant Effect Predictor (VEP, release 87) (16) was used to annotate variants with respect to functional consequences (type of mutation and prediction of the functional impact on the protein by SIFT.2.2 and PolyPhen 2.2.2) and frequencies in public (dbSNP147, 1000 Genomes phase 3, ExAC r3.0, COSMIC v79) and in-house databases. We used the Cancer Genome Interpreter (17) to predict driver and passenger mutations.

***Dendrogram*.** SNV mutations were aggregated as a binary score (1 if a non-synonymous or frameshifting, exonic or on a splice-site was observed, else 0) at the gene level. Resulting profiles were clusterized using the binary distance and Ward's aggregation method. All computation and figure processings were performed using R v4.0.2.

***ADO and false-positive rate estimation*.** CTC and $CD45^{+}$ pool DNA were amplified before sequencing. To estimate ADO, we selected all reliable variants in germline or CD45 DNA using HaplotypeCaller with the following post-filtering: coverage ≥8 in both samples, ≥5 variant reads representing ≥5% of sequenced reads at that position, genotype quality ≥20. We then compared the proportions of normal/variant reads in the germline and CD45 DNA using Fisher's exact test. Variants with a significant difference ($p < 0.05$), a variant allele fraction between 0.2 and 0.8 in germline DNA and <0.1 or >0.9 in the CD45 DNA were considered to have undergone ADO. To estimate the false-positive rates in CTC samples, we divided the number of potentially false-positive events by the number of target bases covered ≥8X in the same sample. We adopted a conservative approach and considered as false-positive all events not found in the TB and the CDX.

***Copy number analysis*.** To identify copy-number alterations (CNAs), we identified germline single-nucleotide polymorphisms (SNPs) in each sample and we calculated the coverage log-ratio (LRR) and B allele frequency (BAF) at each SNP site. Genomic profiles were divided into homogeneous segments by applying the circular binary segmentation algorithm, as implemented in the Bioconductor package *DNAcopy* (18), to both LRR and BAF values. We then used the Genome Alteration Print (GAP) method (19) to determine the ploidy of each sample, the level of

contamination with normal cells and the allele-specific copy number of each segment. Ploidy was estimated as the median copy-number across the genome. Chromosome aberrations were then defined using empirically determined thresholds as follows: gain, copy number > ploidy + 0.5; loss, copy number < ploidy – 0.5; high-level amplification, copy number > ploidy + 2; homozygous deletion, copy number < 0.5. Finally, we considered a segment to have undergone LOH when the copy number of the minor allele was equal to 0. CIRCOS plots were generated using CIRCOS (20).

***Characterization of known copy number changes in CTCs*.** As expected, the log-ratio (LRR) and B allele frequency (BAF) profiles of CTC samples were too noisy to obtain reliable pangenomic copy-number profiles. However, we observed that many chromosome segments displayed allelic imbalances consistent with the presence of chromosome aberrations identified in other samples, in particular PDX and cell line samples. We used these allelic imbalances to detect chromosome aberrations identified in other samples as follows:

1) For each CTC and each chromosome aberration, we counted the number of SNPs with consistent (e.g. BAF > 0.5 in the CTC and tumor samples) and discordant allelic imbalance

2) We used Fisher's exact test to identify chromosome segments with a significant enrichment in consistent SNPs

3) We considered an aberration to be present in a CTC sample if the Fisher test was significant (p-value < 0.05) with ≥80% consistent SNPs.

***Phylogenetic inference*.** Trees were built using a binary presence/absence matrix built from the VAF of each sample (present = VAF > 0). The R Bioconductor package

phangorn v2.3.1 (21) was used to perform the parsimony ratchet method (22), generating unrooted trees. The number of mutations and driver mutations on each branch were then determined by selecting mutations present in all samples downstream the branch.

***CDX-derived cell line establishment and cell culture*.** After resection, tumor fragments were conserved in RPMI 1640 medium (Life Technologies, Carlsbad, CA, USA) and immediately transferred to the laboratory. After two washes in 1X PBS (Life Technologies) and incubation for 10min in a 10 mL 1X PBS solution containing 1:10 penicillin/streptomycin (penicillin 10,000 units/mL, streptomycin 10,000 µg/mL, Life Technologies), tumor fragments were first mechanically dissociated using a scalpel before enzymatic dissociation with the Tumor Dissociation Human Kit (Miltenyi Biotech, Köln, Germany) according to manufacturer’s protocol. Cell suspension was then successively filtered on 100-µm and 40-µm cell strainer and washed with PBS 1X before counting. Depletion of mouse cells was performed with the Mouse Cell Depletion Kit according to manufacturer’s protocol using an AutoMacs Pro Separator (Miltenyi Biotec). Tumor cells were then centrifuged and resuspended in Advanced DMEM/F12 medium (Life Technologies) supplemented with 10% FBS, 1% antibiotics (penicillin-streptomycin) and 1% ultraglutamine (Lonza, Basel, Switzerland). After counting, cells were plated in six-well plates (TPP, Trasadingen, Switzerland) coated with poly-L-lysine (Merck, Sigma-Aldrich LLC., Saint-Louis, MO, USA). For GR-CDXL3 and GR-CDXL4 cells, culture medium is supplemented with ROCK1 inhibitor 10nM (Y-27632; Selleckchem, Houston, TX, USA). Cells were observed three times a week and passaged in tissue culture flasks for cell expansion, freezing or characterization; cells were detached using 0.005% trypsin-EDTA (Life Technologies) before centrifugation and counting. The same

normal-serum culture medium was used for cell expansion and permanent culture. Human adenocarcinoma NSCLC cell lines A549 (derived from primary lung tumor) and H441 (derived from pericardial fluid) were obtained through the CANCER-ID consortium and grown in RPMI 1640 medium. Lung A427 cell line (derived from primary lung tumor) was obtained from the American Type culture Collection (ATCC) and grown in MEM medium (Sigma Aldrich) supplemented with 10% FBS, 1% antibiotics, 1% ultraglutamine and 1% sodium pyruvate (Thermo Scientific). All cell lines were regularly verified for mycoplasma contamination using MycoAlert (Lonza).

***Immunohistochemistry*.** IHC staining was performed on formalin-fixed, paraffin-embedded (FFPE) tissue from patient biopsy specimens, CDX and CDX-derived cell lines with antibodies to CK8/18, EpCAM, Ki67 antigen, Vimentin, CD44, TTF1, p40, CK5/6, Chromogranin A, Synaptophysin and SLFN11 (**Supplementary Table 4**). All primary antibody incubations were carried out at RT for 1h except incubation with CK5/6 and SLFN11, which was performed at 37°C. IHC stains were examined by an experienced pathologist (JYS).

***FACS analysis*.** Aldehyde dehydrogenase (ALDH) activity was performed using the Aldefluor kit (StemCell Technologies) according to manufacturer's instructions. EpCAM, CD133, CD166, pan-cytokeratins and E-cadherin antibodies were used according to manufacturer's protocol. Fixation and permeabilization steps were performed for pan-cytokeratins and E-cadherin antibodies using the Fix&Perm kit (ThermoFisher) according to the manufacturer's instructions. $2x10^5$ cells were incubated with each antibody or corresponding negative control isotype antibodies (**Supplementary Table 5**) at RT for 20 min. Acquisition was performed with LSR

Fortessa cytometer (BD Biosciences) equipped with BD FACS Diva software. Data were analyzed using the Kaluza software (Beckman Coulter).

***Protein extraction and western blot analysis*.** Cells were lysed in 1% NP40 lysis buffer [150 mM NaCl, 1 mM EDTA, 50 mM Tris pH7.5, 0.5% NP40, H_2O] supplemented with protease and phosphatase inhibitors (Roche) for 30min on ice. Protein concentrations were measured using the Micro BCA^{TM} protein assay kit (Thermo Scientific). Laemmli buffer (4X) containing β-mercaptoethanol was added and samples were subsequently denatured by boiling at 95°C. Proteins were resolved by 6, 8 or 10% SDS-polyacrylamide gels and transferred onto a nitrocellulose membrane (Bio-Rad), followed by a PBS-milk (5%)-blocking step (1h). Signals were detected using WesternBright ECL (Advansta) on the ChemiDoc XRS+ System (Bio-Rad). The antibodies used in this study are listed in **Supplementary Table 6**.

***RNA extraction and quantitative real-time PCR analysis*.** Total RNA was isolated using the ReliaPrep RNA Cell Miniprep System (Promega) and 1 µg of RNA was used to synthesize cDNA using the High-capacity RNA-to-cDNA^{TM} kit (Thermo Scientific) according to manufacturer's instructions. The primers used were as follows:

- *SLFN11* (forward 5'-GGCCCAGACCAAGCCTTAAT-3' and reverse 5'-CACTGAAAGCCAGGGCAAAC-3')
- *GAPDH* (forward 5'-CCTCAACGACCACTTTGTCA-3' and reverse 5'-TTCCTCTTGTGCTCTTGCTG-3')

Quantitative real-time PCR was carried out using Maxima SYBR Green/ROX qRT-PCR Master Mix (Thermo Scientific) on the Viia7 PCR system (Applied Biosystems).

Relative gene expression was calculated according to the ΔΔCq method and normalized to *GAPDH* expression.

Drugs and chemicals. Aphidicolin (CAS 38966-21-1, Calbiochem) was purchased from Merck. Cisplatin (Mylan Pharma) and PI3KA inhibitor BYL719 (Novartis) were kind gifts from Dr S. Postel-Vinay and Dr Fabrice Andre (INSERM U981, Gustave Roussy, Villejuif) respectively. The PARPi olaparib (AZD-2281, AstraZeneca) was purchased from Selleck Chemicals. KIFC1 inhibitor AZ82 (AOB4872-5) was obtained from CliniSciences. All stock solutions were prepared in DMSO.

***Stable cell lines expressing mCherry-Luc or GFP-Luc*. ** Stable tumor cell lines were established after infection with retroviral/lentiviral vectors (plasmids: MI-Luc-IRES-mCherry #75020, Addgene Teddington, UK; lentivirus: RediFect^{TM} Red-FLuc-GFP, PerkinElmer). Production and titration of retroviral particles were performed as previously described (23). Infection was performed on retronectin-coated plates (TaKaRa Bio, CA, USA) and efficiency was assayed by testing mCherry expression using flow cytometry. When efficiency was below 98%, cell sorting was performed.

Additional References

1. Imielinski M et al. Mapping the Hallmarks of Lung Adenocarcinoma with Massively Parallel Sequencing. *Cell* 2012;150(6):1107–1120.

2. Rizvi NA et al. Mutational landscape determines sensitivity to PD-1 blockade in non–small cell lung cancer. *Science* 2015;348(6230):124–128.

3. Caso R et al. Preoperative clinical and tumor genomic features associated with pathologic lymph node metastasis in clinical stage I and II lung adenocarcinoma. *npj Precis. Onc.* 2021;5(1):1–8.

4. Jordan NV et al. HER2 expression identifies dynamic functional states within circulating breast cancer cells. *Nature* 2016;537(7618):102–106.

5. Ding L et al. Somatic mutations affect key pathways in lung adenocarcinoma. *Nature* 2008;455(7216):1069–1075.

6. Pailler E et al. Acquired Resistance Mutations to ALK Inhibitors Identified by Single Circulating Tumor Cell Sequencing in ALK-Rearranged Non–Small-Cell Lung Cancer. *Clin Cancer Res* 2019;25(22):6671–6682.

7. Faugeroux V et al. An Accessible and Unique Insight into Metastasis Mutational Content Through Whole-exome Sequencing of Circulating Tumor Cells in Metastatic Prostate Cancer. *European Urology Oncology* 2020;3(4):498–508.

8. Polzer B et al. Molecular profiling of single circulating tumor cells with diagnostic intention. *EMBO Mol Med* 2014;6(11):1371–1386.

9. Martin M. Cutadapt removes adapter sequences from high-throughput sequencing reads. *EMBnet.journal* 2011;17(1):10–12.

10. Khandelwal G et al. Next-Generation Sequencing Analysis and Algorithms for PDX and CDX Models. *Mol Cancer Res* 2017;15(8):1012–1016.

11. Li H, Durbin R. Fast and accurate short read alignment with Burrows–Wheeler transform. *Bioinformatics* 2009;25(14):1754–1760.

12. Tarasov A, Vilella AJ, Cuppen E, Nijman IJ, Prins P. Sambamba: fast processing of NGS alignment formats. *Bioinformatics* 2015;31(12):2032–2034.

13. DePristo MA et al. A framework for variation discovery and genotyping using next-generation DNA sequencing data. *Nat Genet* 2011;43(5):491–498.

14. Auwera GAV der et al. From FastQ Data to High-Confidence Variant Calls: The Genome Analysis Toolkit Best Practices Pipeline. *Current Protocols in Bioinformatics* 2013;43(1):11.10.1-11.10.33.

15. Cibulskis K et al. Sensitive detection of somatic point mutations in impure and heterogeneous cancer samples. *Nat Biotechnol* 2013;31(3):213–219.

16. McLaren W et al. The Ensembl Variant Effect Predictor. *Genome Biology* 2016;17(1):122.

17. Tamborero D et al. Cancer Genome Interpreter annotates the biological and clinical relevance of tumor alterations. *Genome Medicine* 2018;10(1):25.

18. Venkatraman ES, Olshen AB. A faster circular binary segmentation algorithm for the analysis of array CGH data. *Bioinformatics* 2007;23(6):657–663.

19. Popova T et al. Genome Alteration Print (GAP): a tool to visualize and mine complex cancer genomic profiles obtained by SNP arrays. *Genome Biology* 2009;10(11):R128.

20. Krzywinski M et al. Circos: An information aesthetic for comparative genomics. *Genome Res.* 2009;19(9):1639–1645.

21. Schliep KP. phangorn: phylogenetic analysis in R. *Bioinformatics* 2011;27(4):592–593.

22. Nixon KC. The Parsimony Ratchet, a New Method for Rapid Parsimony Analysis. *Cladistics* 1999;15(4):407–414.

23. Hamelin V, Letourneux C, Romeo P-H, Porteu F, Gaudry M. Thrombopoietin regulates IEX-1 gene expression through ERK-induced AML1 phosphorylation. *Blood* 2006;107(8):3106–3113.

Abbreviations

CTC: circulating tumor cell

CDX: CTC-derived eXplant

NSCLC: non-small cell lung cancer

DDR: DNA damage response

HR: homologous recombination

HRD: homologous recombination deficiency

NHEJ: non-homologous end joining

PARPi: poly-ADP polymerase inhibitor

TB: tumor biopsy

NB: nuclear body

CAM: chick embryo chorioallantoic membrane

CNA: copy number analysis

CIN: chromosomal instability

WGD: whole-genome doubling

WES: whole-exome sequencing

IHC: immunohistochemistry

RT: room temperature

BLI: bioluminescence imaging

Supplementary Figure 1

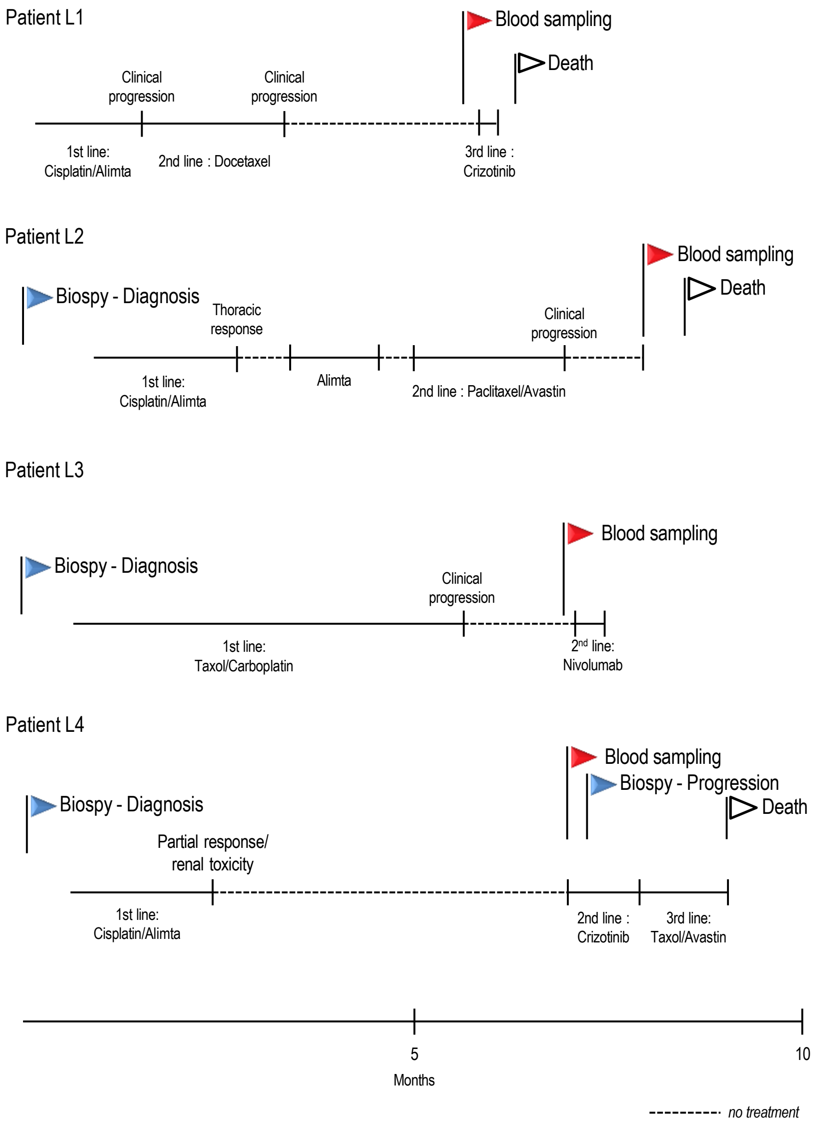

Supplementary Figure 2

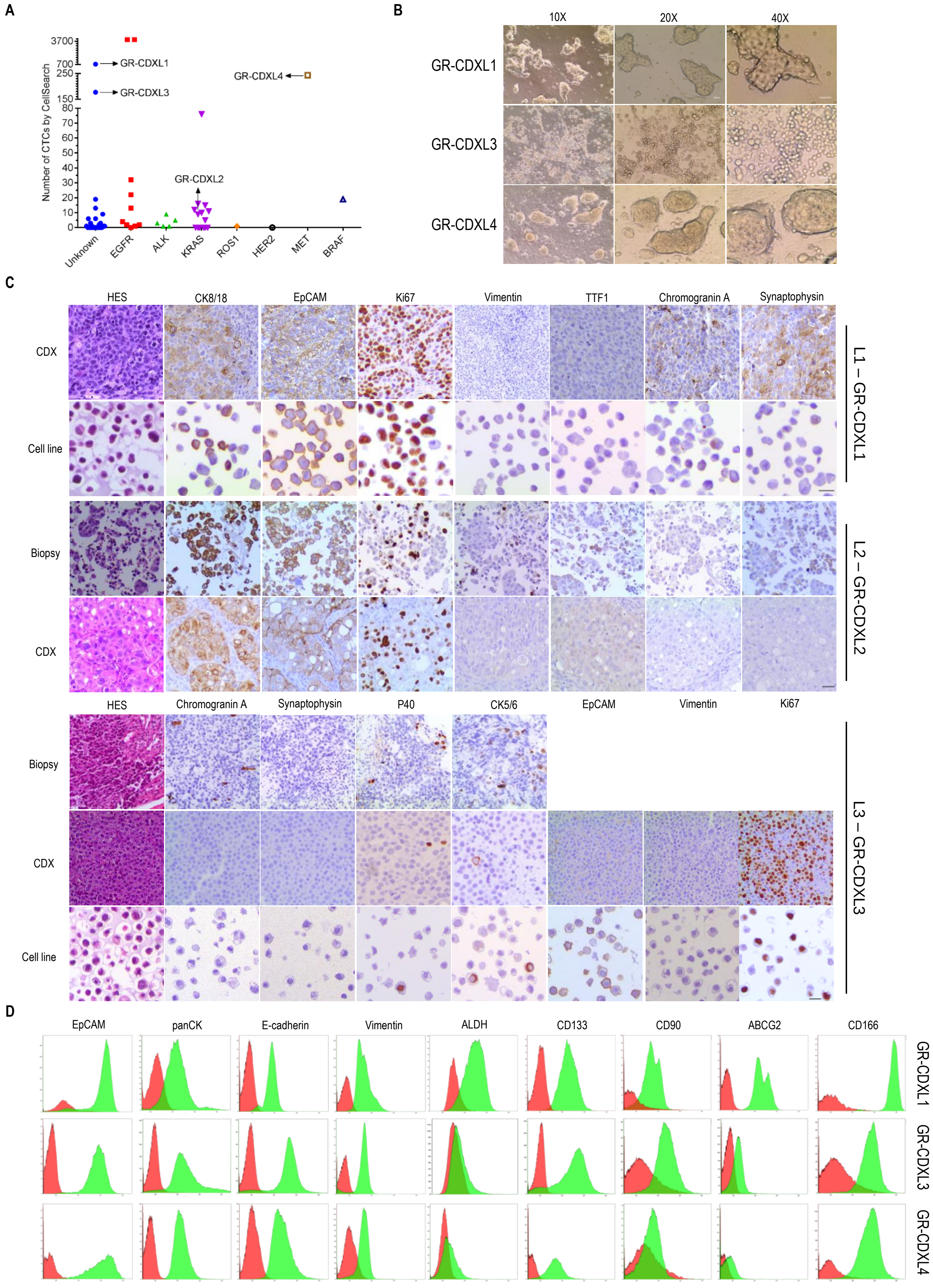

Supplementary Figure 3

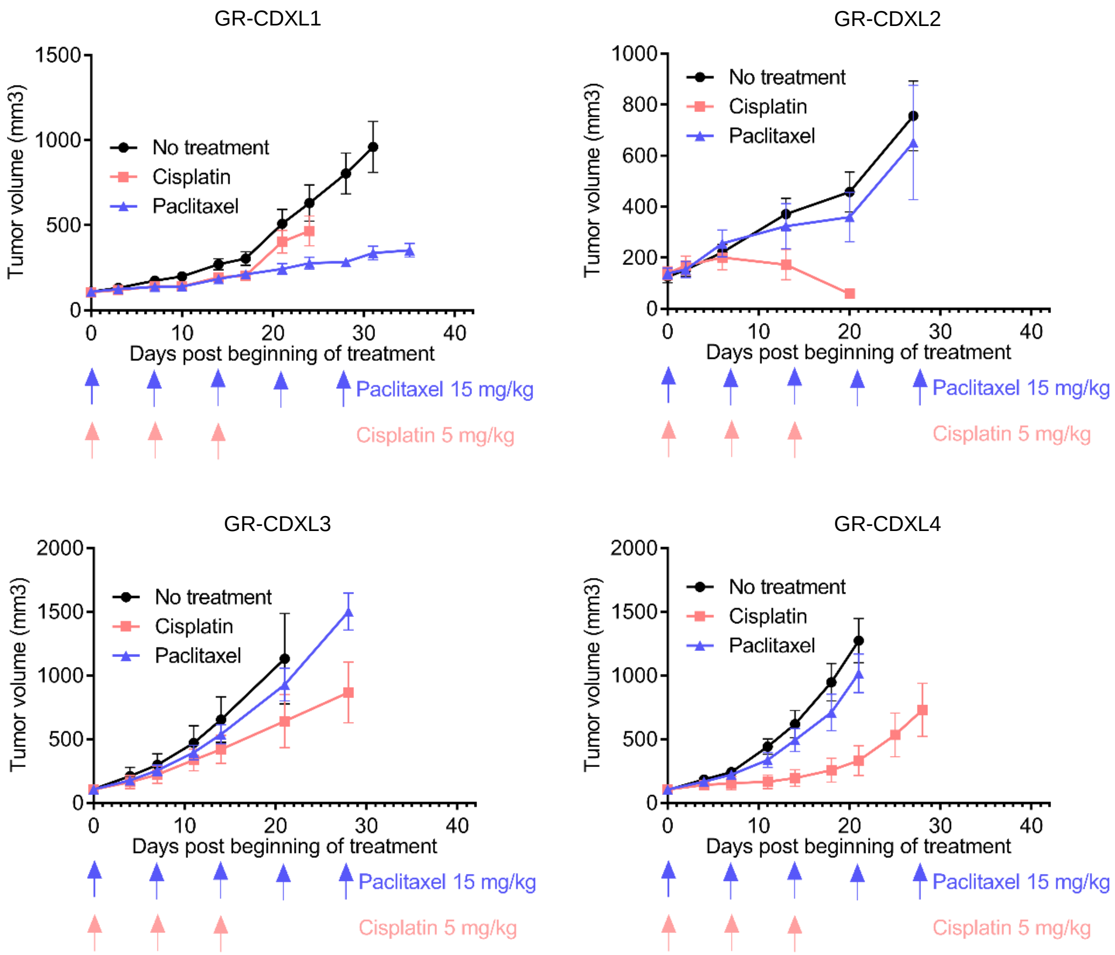

Supplementary Figure 4

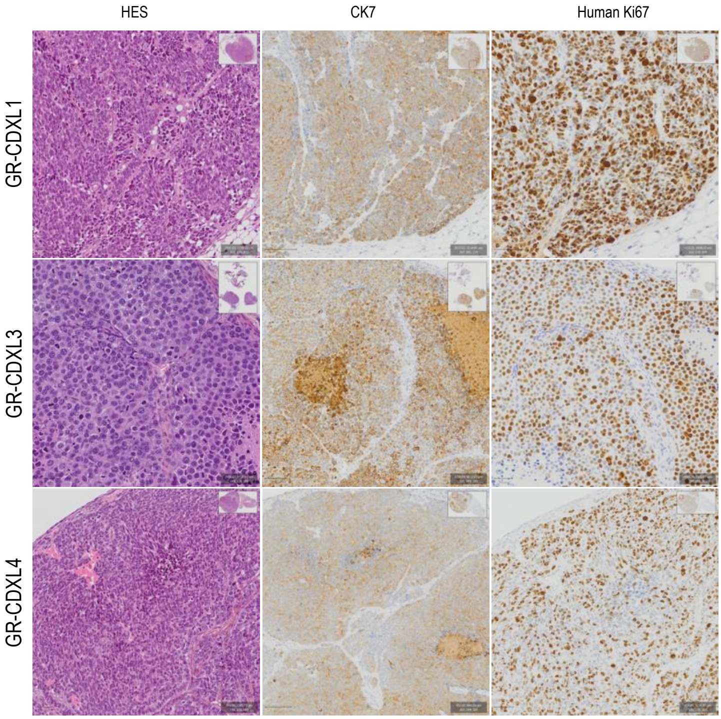

Supplementary Figure 5

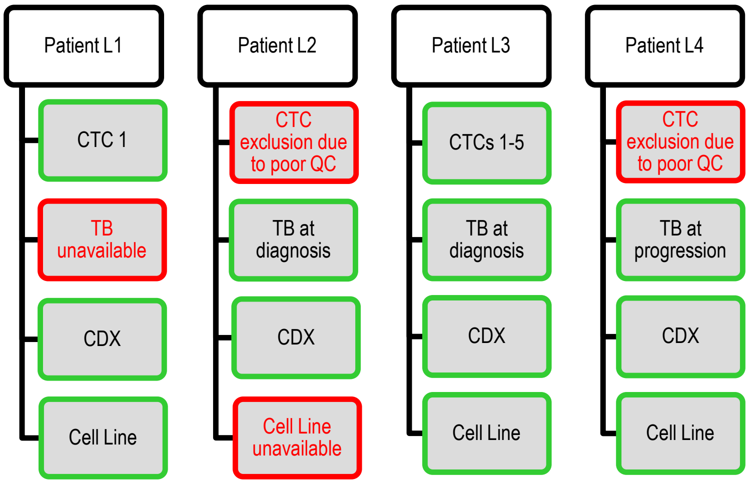

Supplementary Figure 6

A

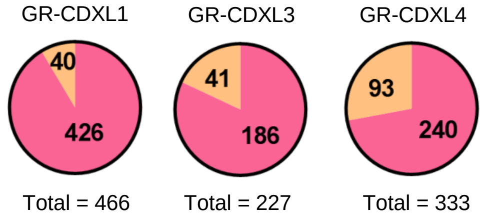

B

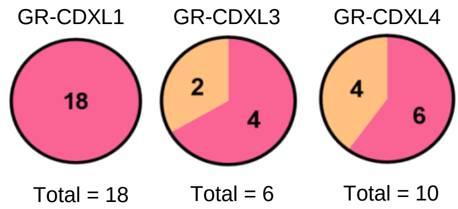

C

GR-CDXL1

SPEN	D3584H
ARID1A	S696X
ARID1A	1711-1712X
ABL2	Y486F
ABL2	D371Y
TP53	R196P
SETBP1	V1430L
RHOA	E40Q
ARID1B	M874K
KMT2C	E4543K
UBR5	G2560C
ATRX	V1568F
EIF4G3	D1165H
BRCA2	H3010Y
TP53BP1	S207C
SOX9	V115L
EPHA4	R209C
PIK3R4	R103W

GR-CDXL3

TP53	Y163C
ASPM	A999S
DDB1	G1035D
HERC2	G4018D

GR-CDXL4

ATP1A1	S452C
TP53	M237I
NF1	E479
ACVR1	W461C
RB1	E539
DLC1	A1268P

D

GR-CDXL3

**HERC2**	G4018D
**MED23**	K359M

GR-CDXL4

**CIC**	L1350V
**ACVR1**	W461C
**MLLT4**	P1299T
**PTGS1**	E338K

Shared by CDX/CL
Only in CDX

Supplementary Figure 7

A

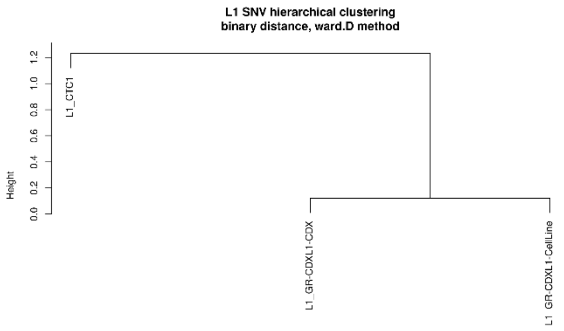

B

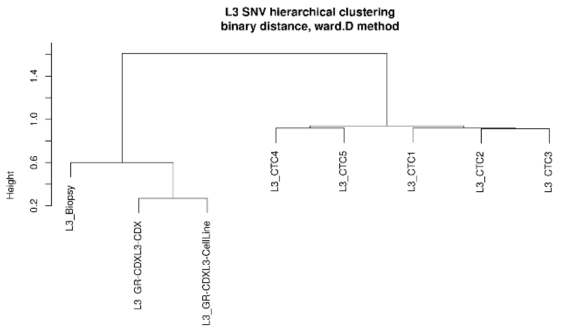

C

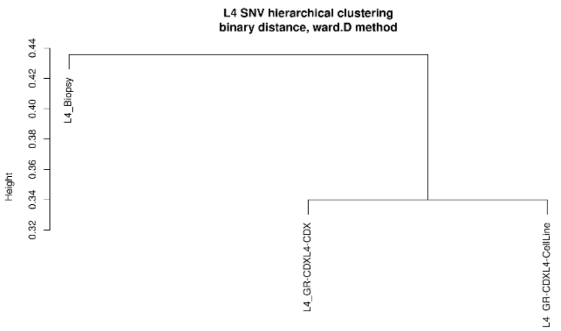

Supplementary Figure 8

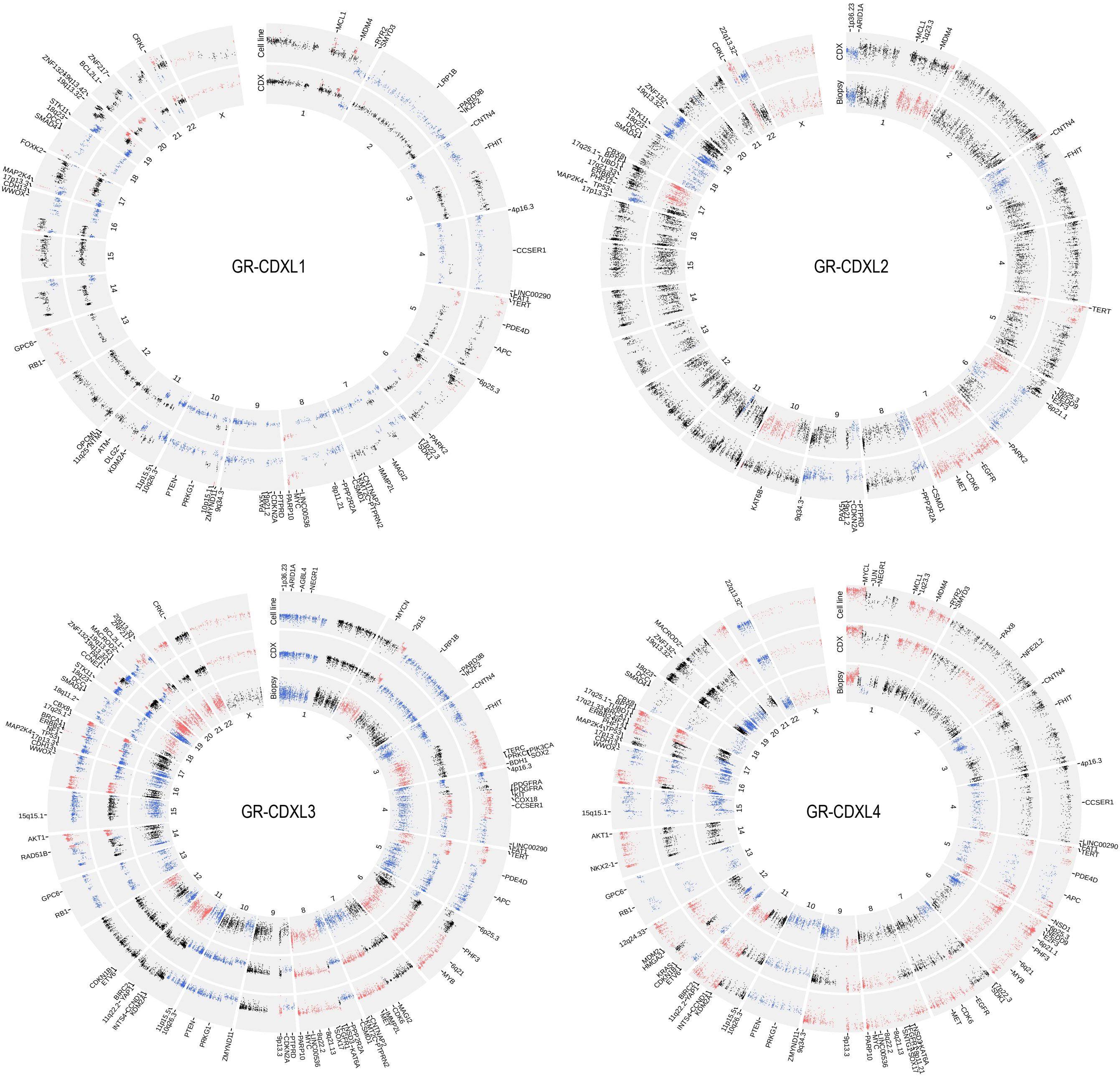

Supplementary Figure 9

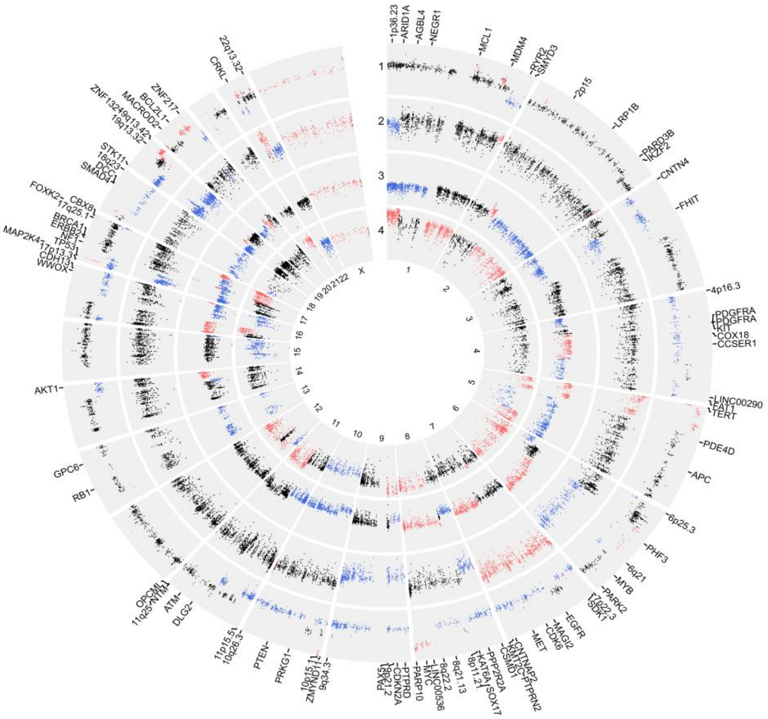

Supplementary Figure 10

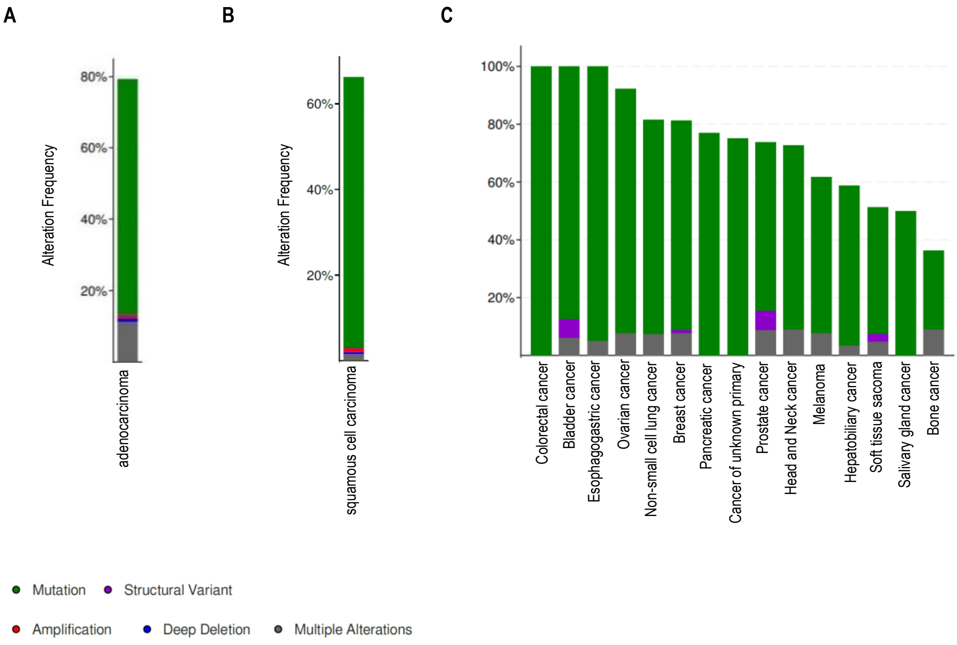

Supplementary Figure 11

A

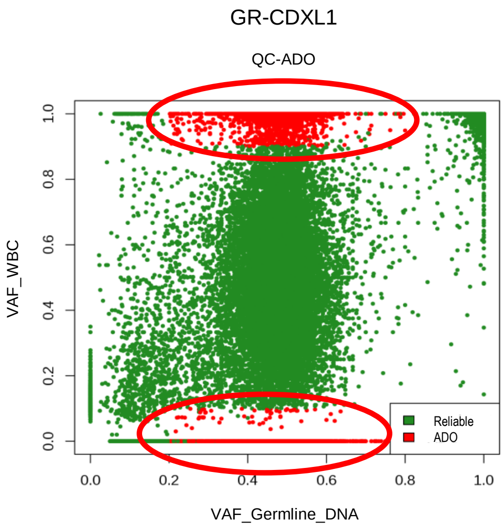

Proportion of germline variants with ADO
0,157515487

Sample	Number of false-positive variants	Number of target bases covered ≥8X	False-positive rate
L1-CTC1	1306	10465312	0,0001247

B

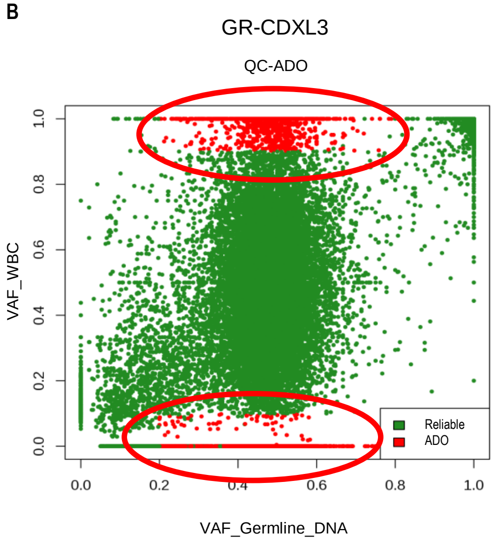

Proportion of germline variants with ADO
0,09321979

Sample	Number of false-positive variants	Number of target bases covered ≥8X	False-positive rate
L3-CTC1	1647	35136861	4,68739E-05
L3-CTC2	1396	29505812	4,73127E-05
L3-CTC3	1599	32123746	4,97763E-05
L3-CTC4	1295	36725416	3,52617E-05
L3-CTC5	932	32677245	2,85214E-05

Supplementary Figure 12

A

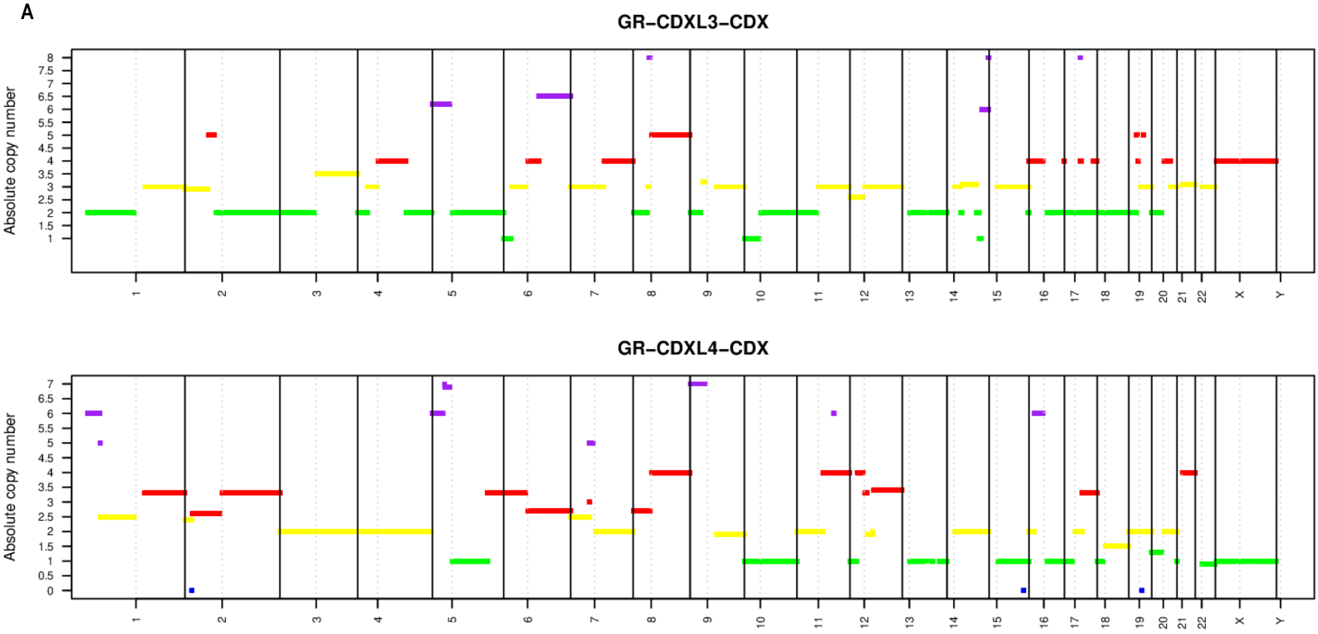

B

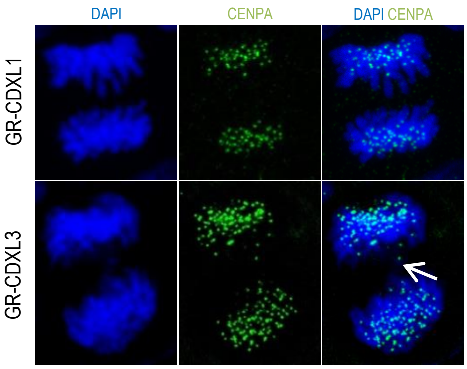

C

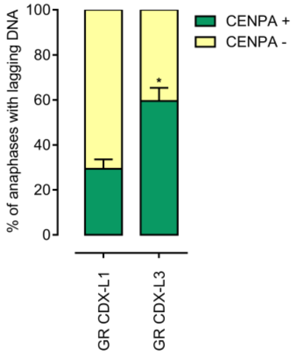

Supplementary Figure 13

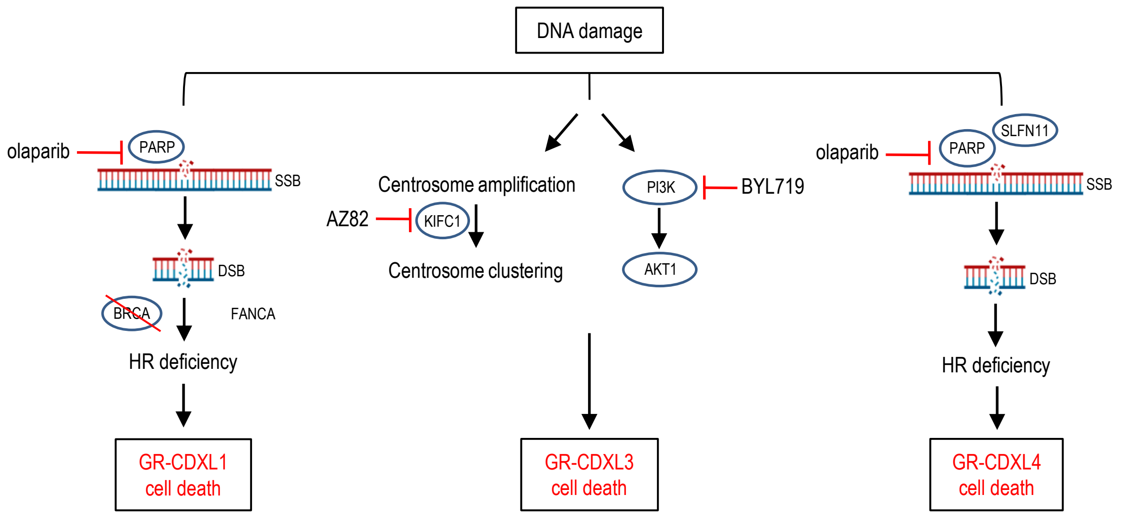

Supplementary Figure 14

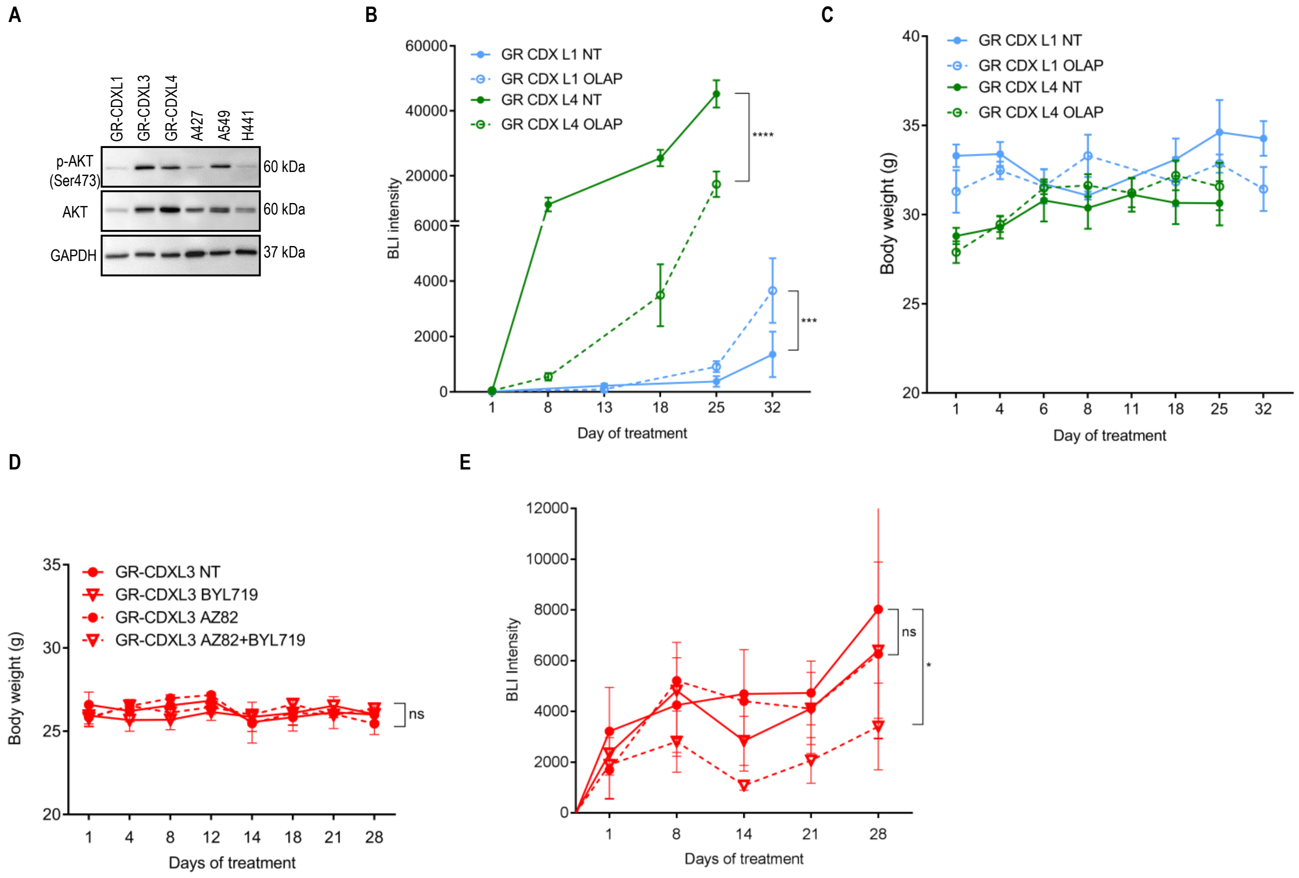

Supplement: Supplemental data [file jciinsight-7-155804-s144.pdf]
