# Supplementary material for: Targeting genome integrity dysfunctions impedes metastatic potency in non–small cell lung cancer circulating tumor cell–derived explants
Source: JCI Insight. 2022 Jun 8;7(11):e155804. doi: 10.1172/jci.insight.155804 (PMC9220846; doi:10.1172/jci.insight.155804)
Supplement: Supplemental tables 1-7 [file jciinsight-7-155804-s151.pdf]

Supplementary Table 1

Patient	Age	Gender	Histology	Smoking status (PY)	# lines of therapy	# metastatic sites	Oncogenic drivers	# CTCs by CS /7.5 mL blood	# CTCs injected	Model
P1	45	F	Adenocarcinoma	nonsmoker	6	4	-	9	23	
P2	52	M	Adenocarcinoma	25	4	1	*BRAF*	1	3	
P3	64	F	Adenocarcinoma	nonsmoker	4	3	*EGFR*	22	62	
P4	53	M	Adenocarcinoma	40	1	1	*KRAS*	5	17	
P5	40	M	Adenocarcinoma	20	0	2	-	ND	ND	
P6	62	F	Adenocarcinoma	nonsmoker	1	3	*EGFR*	0	0	
P7	72	M	Adenocarcinoma	1	7	0	*KRAS*	0	0	
P8 (termed L1)	39	M	Adenocarcinoma	50	2	2	-	750	3500	GR-CDXL1
P9	37	M	Large cell carcinoma	4-5	1	2	*ROS1*	1	5	
P10	61	F	Adenocarcinoma	35	7	1	*KRAS*	0	0	
P11	55	M	Adenocarcinoma	20	1	2	-	0	0	
P12	51	M	Large cell carcinoma	50	1	0	-	0	0	
P13	64	M	Adenocarcinoma	45	1	3	*KRAS*	0	0	
P14	55	M	Adenocarcinoma	10	2	1	*ALK*	9	38	
P15	56	M	Adenocarcinoma	*	1	3	*BRAF*	0	0	
P16	62	F	Adenocarcinoma	25	6	2	*KRAS*	0	0	
P17	66	F	Adenocarcinoma	nonsmoker	2	2	*EGFR*	2	9	
P18	46	F	Adenocarcinoma	nonsmoker	1	1	*EGFR*	32	137	
P19	42	F	Adenocarcinoma	20	2	2	*c-MET*	1	4	
P20	61	F	Adenocarcinoma	35	3	2	-	3	13	
P21	74	M	Adenocarcinoma	80	2	4	-	0	0	
P22	61	M	Adenocarcinoma	60	4	3	*ALK*	5	19	
P23	69	M	Squamous cell carcinoma	50	2	0	-	0	0	
P24	68	M	Adenocarcinoma	100	0	3	-	2	8	
P25	52	F	Adenocarcinoma	32	3	3	*KRAS*	15	62	
P26	56	F	Adenocarcinoma	35	1	2	*KRAS*	76	253	
P27	56	F	Squamous cell carcinoma	40	5	1	-	1	4	
P28	65	M	Adenocarcinoma	30	2	1	-	6	22	
P29	58	M	Adenocarcinoma	50	4	1	*ALK*	1	4	
P30	65	M	Adenocarcinoma	15	1	3	*KRAS*	12	42	
P31	55	F	Adenocarcinoma	7	5	0	*ALK*, *MET*	1	4	
P32	66	M	Adenocarcinoma	8	1	1	*EGFR*	13	49	
P33	64	M	Adenocarcinoma	5	0	2	*EGFR*	4	17	
P34	65	M	Adenocarcinoma	70	1	1	-	0	0	
P35	59	M	Squamous cell carcinoma	15	1	3	-	19	86	
P36	56	M	Squamous cell carcinoma	40	3	4	-	6	19	
P37 (termed L2)	45	F	Adenocarcinoma	25	2	3	*KRAS*	10	35	GR-CDXL2
P38	53	M	Adenocarcinoma	40	1	2	*KRAS*	16	66	
P39	81	F	Adenocarcinoma	nonsmoker	2	3	*EGFR*	3903	17694	
P40	63	M	Squamous cell carcinoma	40	0	3	-	0	0	
P41	59	M	Adenocarcinoma	unknown	1	3	-	0	0	
P42	71	F	Adenocarcinoma	2	6	1	*EGFR*	1	4	
P43	51	M	Adenocarcinoma	18	1	3	*EGFR*	13	54	
P44	59	F	Adenocarcinoma	nonsmoker	0	1	-	1	4	
P45	40	F	Adenocarcinoma	15	0	1	*KRAS*	9	47	
P46	67	M	Squamous cell carcinoma	1	2	2	-	0	0	
P47	54	M	Adenocarcinoma	30	1	1	*HER2*	0	0	
P48 (termed L3)	55	M	Squamous cell carcinoma	34	1	3	-	117	330	GR-CDXL3
P49	69	F	Adenocarcinoma	nonsmoker	2	1	*ALK*	3	16	
P50 (termed L4)	66	F	Adenocarcinoma	80	1	3	*MET*	243	1102	GR-CDXL4
P51	58	M	Adenocarcinoma	35	1	2	*BRAF*	19	86	
P52	71	F	Adenocarcinoma	nonsmoker	3	1	*EGFR*	2	8	
P53	53	M	Adenocarcinoma	40	2	3	*KRAS*	0	0	
P54	64	M	Adenocarcinoma	40	2	1	*KRAS*	0	0	
P55	73	M	Large cell carcinoma	60	2	4	-	0	0	

Supplementary Table 2

Model	Sample	Mean Depth	Coverage above 25X	SNVs	INDELs	SNVs+INDELs
GR-CDXL1	L1-CDX	158	97	450	17	467
	L1-CellLine	154	97	436	14	450
GR-CDXL2	L2-TB	148	91	363	217	580
	L2-CDX	110	81	436	20	456
GR-CDXL3	L3-TB	73	92	287	7	294
	L3-CDX	97	84	232	10	242
	L3-CellLine	96	84	221	6	227
GR-CDXL4	L4-TB	110	96	282	13	295
	L4-CDX	114	88	332	11	343
	L4-CellLine	112	87	263	11	274

Supplementary Table 3

Model	Sample	Mean Depth	Coverage above 25X	SNVs+INDELs shared by at least 2 CTCs	SNVs+INDELs shared by at least 1 CTC and TB	SNVs+INDELs shared by at least 1 CTC and CDX	High-confidence SNVs+INDELs*
GR-CDXL1	L1-CTC1	32	11	N/A	N/A	24	24
GR-CDXL3	L3-CTC1	73	48	418	39	36	41
	L3-CTC2	50	39	78	57	58	64
	L3-CTC3	54	43	95	64	67	70
	L3-CTC4	76	50	72	71	69	78
	L3-CTC5	76	45	60	51	50	55

*Unique SNVs and INDELs were obtained by adding SNVs and INDELs shared by at least 1 CTC and TB and SNVs and INDELs shared by at least 1 CTC and the CDX. Duplicate values were counted only once.

Supplementary Table 4

Antibody	Manufacturer	Reference	Clone	Species	Dilution	Antigen retrieval
CK8/18	Novocastra	#NCL-L-CK5/6/8/18	5D3, LP34	mouse	1/100	40 minutes
CK5/6	DAKO	#M7237	D5/16B4	mouse	1/10	40 minutes
CK7	DAKO	#M7018	OV-TL 12/30	mouse	1/50	20 minutes
EpCAM	Cell Signaling	#2929S	VU1D9	mouse	1/500	40 minutes
Ki67	DAKO	#M7240	MIB-1	mouse	1/20	20 minutes
Vimentin	Santa Cruz	#SC-6260	V9	mouse	1/500	No retrieval
TTF1	DAKO	#M3575	8G7G3/1	mouse	1/50	60 minutes
Chromogranin A	DAKO	#M0869	DAK-A3	mouse	1/50	40 minutes
Synaptophysin	DAKO	#M7315	DAK-SYNAP	mouse	1/16	40 minutes
P40	DBS	#RP 163-05	polyclonal	rabbit	1/50	40 minutes
SLFN11	Cell Signaling	#34858	D8W1B	rabbit	1/50	20 minutes

Supplementary Table 5

Antibody	Manufacturer	Reference	Clone	Species	Fluorochrome	Dilution	Isotypes
EpCAM	BD Pharmingen	347200	EBA-1	mouse	APC	1/20	Ms IgG1
CD133-2	Miltenyi	130-098-046	293C3	mouse	PE	1/10	Ms IgG2b
CD90	BD Pharmingen	555596	5E10	mouse	PE	1/5	Ms IgG1
ABCG2	R&D system	FAB995P	5D3	mouse	PE	1/5	Ms IgG2b
CD166	R&D system	FAB6561P	105902	mouse	PE	1/100	Ms IgG1
CD24	BD Pharmingen	555428	ML5	mouse	PE	1/5	Ms IgG2a
CD44	BD Pharmingen	555478	G44-26	mouse	FITC	1/5	Ms IgG2b
Pan-cytokeratins	ebioscience	53-9003-82	AE1/AE3	mouse	AF488	1/100	Ms IgG1
E-cadherin	BD Pharmingen	560061	36/E-Cadherin	mouse	AF488	1/200	Ms IgG2a
Vimentin	Santa Cruz	sc-6260	V9	mouse	AF488	1/2	Ms IgG1

Supplementary Table 6

Antibody	Manufacturer	Reference	Clone	Species	Dilution
p-CHK1 (Ser345)	Cell Signaling	#2348	133D3	rabbit	1/500
p-AKT (Ser473)	Cell Signaling	#9271	polyclonal	rabbit	1/1000
CHK1	Santa Cruz	sc-8408	G-4	mouse	1/500
AKT	Cell Signaling	#4685	11E7	rabbit	1/1000
FANCA	Bethyl Lab	A301-980A	polyclonal	rabbit	1/500
PARP1	Cell Signaling	#9542	polyclonal	mouse	1/1000
SLFN11	Santa Cruz	sc-374339	E-4	mouse	1/500
GAPDH	Santa Cruz	sc-47724	0411	mouse	1/1000

Supplementary Table 7

Antibody	Manufacturer	Reference	Clone	Species	Dilution
Cyclin A	Santa Cruz	sc-271682	B-8	mouse	1/300
53BP1	Abcam	ab21083	polyclonal	rabbit	1/300
H2AX (Ser139)	Merck	05-636	JBW301	mouse	1/3000
Geminin	Santa Cruz	sc-74456	F-7	mouse	1/300
RAD51	Merck	PC130	polyclonal	rabbit	1/300
p-RPA32 (S33)	Bethyl Lab	A300-246A-M	polyclonal	rabbit	1/1000
p-DNAPK	Abcam	ab18356	10B1	mouse	1/1000
CENPA	Abcam	ab13939	3-19	mouse	1/1000
